# Multimechanistic actions of functional factors in enhancing physical strength and endurance: a scoping review of nutritional basis and natural extracts

**DOI:** 10.3389/fspor.2026.1819117

**Published:** 2026-05-13

**Authors:** Xiangyu Huang, Jingjing Zhu, Peng Zang, Junli Chen, Pu Chen, Nan Xu, Wei Liu

**Affiliations:** 1 College of Food Science and Engineering, Henan University of Technology, Zhengzhou, China; 2 China Astronaut Research and Training Center, Beijing, China

**Keywords:** endurance enhancement, functional factors, natural extracts, physical strength enhancement, sports nutrition

## Abstract

This article provides a scoping review of various types of functional factors and their mechanisms of action in enhancing physical strength and endurance, focusing on their roles in regulating the central nervous system, improving energy metabolism, reducing metabolite accumulation, enhancing antioxidant and anti-inflammatory capacity, regulating the endocrine system, and improving blood circulation. For functional factors supported by sufficient human evidence, stratified application can be made according to their primary effects. Those applicable to strength-type sports include creatine, HMB, vitamin D, L-arginine, L-citrulline, L-carnitine, curcumin, epicatechin, and golden root extract. Those applicable to endurance-type sports include beta-alanine, taurine, iron, caffeine, eleutheroside, anthocyanins, powdered Montmorency tart cherry, and beetroot concentrate. Those applicable to mixed-discipline sports include BCAAs, glutamine, whey protein, coenzyme Q10, and magnesium. Mechanisms that specifically enhance physical strength include increased blood flow delivery via the NO–sGC–cGMP pathway, attenuation of central nervous system fatigue through LAT1-mediated tryptophan competition, and maintenance of calcium homeostasis via the SERCA–RyR1–VDAC1–MCU axis. Endurance-specific enhancement is achieved primarily by promoting type IIa muscle fiber formation through the AMPK/SIRT1/PGC1-α and PKA–CREB–ERRγ signaling axes. Functional factors that simultaneously improve both outcomes act through reducing oxidative stress via the Nrf2–Keap1–ARE network, regulating energy metabolism via the PI3K/Akt/mTOR and AMPK pathways, suppressing NF-κB-mediated inflammation, and optimizing the gut–liver–muscle axis. In addition, this article points out research challenges such as individual differences and experimental design limitations and suggests that the long-term safety of personalized sports nutrition strategies and functional factors needs to be further explored in the future. The review provides a scientific theoretical basis for sports training and health management, as well as an important reference for the development of novel sports nutrition programs.

## GRAPHICAL ABSTRACT

**Figure:**
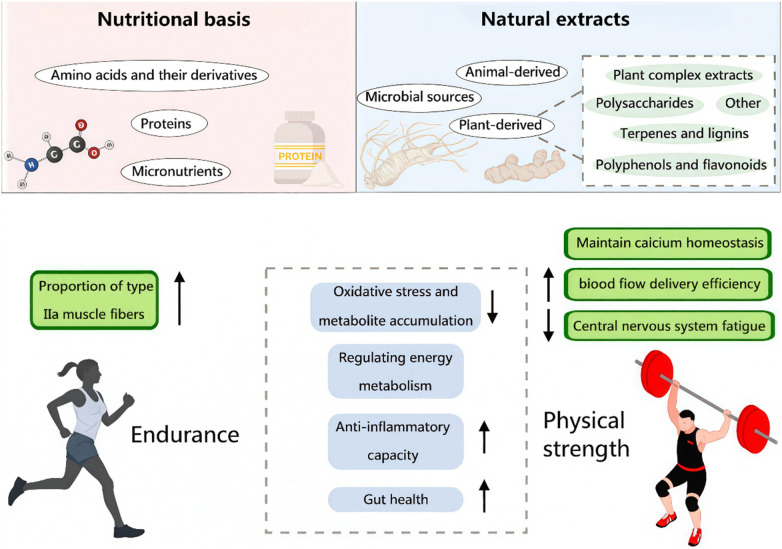
This graphical abstract categorizes functional factors into two distinct categories, which are basic nutritional components and natural extracts derived from animal, plant and microbial sources. It further clarifies three categories of action mechanisms of these functional factors. The first category is the specific mechanisms for enhancing physical strength, which include the maintenance of calcium homeostasis, improvement of blood flow efficiency, as well as the alleviation of central nervous system fatigue. The second category is the specific mechanism for improving endurance via increasing the proportion of type IIa muscle fibres. The third category is the shared mechanisms that boost both physical strength and endurance, including reducing oxidative stress and metabolite accumulation, regulating energy metabolism, enhancing anti-inflammatory capacity, and improving gut health.

## 1 Introduction

Endurance and physical strength are important indicators for exercise level and health. Physical strength refers to the ability of the human body to withstand physical activities or labor, focusing on muscular strength and explosive power. Endurance refers to the ability to maintain a specific intensity of exercise, including cardiorespiratory endurance and muscular endurance, which focuses on improving aerobic metabolism and prolonging the duration of exercise. In competitive sports, physical endurance determines the performance and stability of athletes during high-intensity exercise or prolonged competitions. In daily life, it also promotes the maintenance of a healthy body, reduces the risk of chronic disease, and improves the quality of life of the elderly or general fitness population.

This paper adopts a scoping review approach to comprehensively search and screen relevant studies, covering all research types from basic experiments to clinical trials. Literature searches were conducted using major databases such as PubMed, Web of Science, and ScienceDirect. The aim of this paper is to systematically summarize and analyze the role of various functional factors and their mechanisms in enhancing physical endurance. The research results of multiple functional factors, not only focusing on their effects on physical endurance, but also exploring the underlying physiological mechanisms, are comprehensively integrated by this review. We will focus on the effects of these functional factors on the central nervous system, improvement of energy metabolism, reduction of metabolite accumulation, antioxidant and anti-inflammatory effects, regulation of the endocrine system, and improvement of blood circulation. The in-depth analysis of the complex physiological and biochemical regulatory network of the body can provide a scientific theoretical basis for sports training and health management.

## 2 Nutritional basis: amino acids, proteins, and micronutrients

The role of various types of functional factors in enhancing physical endurance has attracted widespread attention in recent years. These functional factors can be categorized into nutritional basis and natural extracts. These nutrients are often found in various dietary structures and belong to the category of “daily intake.” They act on the body through different physiological mechanisms and may provide new solutions for improving physical endurance.

### 2.1 Amino acids and their derivatives

Amino acids are amphoteric organic compounds that contain basic amino groups and acidic carboxyl groups, serving as the fundamental units of proteins. Amino acids are primarily classified into essential amino acids and non-essential amino acids. In addition, amino acids can be converted into derivatives such as creatine, which is produced from the reaction of arginine and glycine. Essential amino acids are beneficial for promoting muscle protein synthesis. Branched-chain amino acids (BCAAs) consist of three essential amino acids and can simultaneously enhance physical endurance and strength. BCAAs can promote protein synthesis by activating mTORC1, facilitate fat oxidation by increasing the concentration of intermediates in the tricarboxylic acid cycle, and enhance the activation of S6K while inhibiting 4E-BP1 (1). In addition, BCAAs can prolong exercise endurance (2), enhance squat and bench press strength (3), and increase peak and average power output by reducing the transport of free tryptophan to the brain, thereby decreasing 5-hydroxytryptamine (5-HT) synthesis in the brain (4).

Some non-essential amino acids have significant effects on enhancing physical strength, and these are L-citrulline (LC), L-arginine (L-Arg), and glutamine. LC and L-Arg can promote the production of endogenous nitric oxide, leading to relaxation and dilation of the vascular smooth muscle (5) and reducing oxygen consumption and ATP cost of exercise (6), thereby enhancing maximum grip strength (7) and peak power output (8). Glutamine, as a precursor to the antioxidant glutathione, indirectly exerts antioxidant effects (9). By promoting glycogen synthesis (10) and reducing metabolite accumulation (11), it enables individuals who take it as a supplement to exhibit lower soreness scores and higher peak torque during exercise (12). Furthermore, some amino acid derivatives exhibit similar effects. Studies on creatine have shown that supplementation can enhance lower limb explosive strength (13), potentially due to the effective conversion of ADP to ATP by creatine phosphate, improving the hydration environment within muscle cells and enhancing the activity and differentiation of satellite cells (14). β-Hydroxy-β-methylbutyrate (HMB), derived from the metabolism of leucine, can enhance upper limb strength and reduce muscle damage (15), and it has a similar effect in elderly patients with muscle atrophy (16). The mechanism may involve the activation of the mTOR1 pathway (17) and the inhibition of the ubiquitin–proteasome pathway and caspase activity (18), which increases protein synthesis and decreases degradation, as well as improving energy metabolism through the promotion of fat oxidation. L-carnitine is a type of amino acid that increases fat metabolism by promoting the transport of long-chain fatty acids to mitochondria for oxidation, thereby increasing the number of repetitions and weight in leg press exercises (19), and it also positively impacts the muscle strength of the elderly (20).

For endurance enhancement, among non-essential amino acids, only β-alanine has this effect. Studies indicate that β-alanine can increase carnosine levels, reduce postexercise blood lactate levels, enhance recovery capacity from high-intensity exercise, and improve endurance (21). Long-term supplementation can also elevate peak oxygen consumption and repetitions, while decreasing fatigue index (22). In amino acid derivatives, both taurine and N-acetylcysteine (NAC) have effects on merely extending endurance. Taurine is synthesized from methionine, cysteine, and other compounds. Taurine can extend exercise duration, increase sweating rate and peak oxygen consumption, and reduce perceived exertion and core temperature by promoting cross-bridge formation and muscle contraction (23), thereby enhancing short-term endurance performance (24). NAC, derived from L-cysteine, can prolong endurance through intravenous administration of high doses, which alleviates the increase in plasma K^+^ concentration (25, 26). This may due to its antioxidant properties that can protect the function of Na^+^/K^+^ pumps and ion channel proteins. The studies of amino acids and their derivatives and their effects and mechanisms on enhancing physical strength and endurance are presented in Table 1.

**Table 1:** The studies of amino acids and their derivatives and their effects and mechanisms on enhancing physical strength and endurance.

Active ingredients	Source	Subject/model	Dosage and duration	Exercise/assessment type	Effect	Mechanism	References
BCAAs	Meat, fish, dairy products, and eggs	Volunteers	300 mg kg^−1^ day^−1^ for 3 days	Aerobic endurance	Exercise endurance increased by 17.2%, and fat oxidation increased	Promotes fat oxidation, enhances protein synthesis, inhibits its breakdown, and reduces the formation of fatigue-related neurotransmitters	(2)
		Male athletes	14 g day^−1^ for 56 days	Anaerobic strength (1-RM squat, bench press)	Increases 1-RM squat and bench press strength		(3)
		Male cyclists	12 g day^−1^ for 70 days	Anaerobic power (peak and average power)	Peak power increased by 19%, and average power increased by 4%		(4)
Glutamine	Beans, spinach, cabbage, parsley, wheat, soybeans, eggs, dairy products, yogurt, and cheese	Males and females	0.3 g kg^−1^ day^−1^ for 3 days	Strength/recovery (peak torque at 180°/s)	Lower pain scores and higher peak torque generated at 180°/sec during the muscle recovery process	Enhances immunity, reduces inflammation, and decreases the accumulation of metabolites	(12)
		Professional basketball players (male)	6 g day^−1^ for 20 days	Aerobic endurance (fatigue metabolite monitoring)	Reduction of fatigue metabolite indicators		(11)
L-Arg	Meat, fish, dairy products, and eggs	Athletes (sex not specified)	2 g day^−1^ for 45 days (randomized)	Aerobic capacity (VO₂max, output power)	Maximum oxygen consumption and output power increase	By increasing the levels of the urea cycle, the metabolism of ammonia in the blood is enhanced, thereby enhancing mitochondrial biogenesis and increasing protein synthesis	(7)
LC	Various types of melons	Female adults	8 g, single acute dose	Anaerobic power (peak and burst power)	Peak power and burst power increase		(8)
Creatine	Red meat (beef, lamb), fish, chicken, and animal organs (liver, kidneys)	Athletes (sex not specified)	20 g day^−1^ for 6 days +2 g pre-experiment	Anaerobic strength and sprint (1-RM half squat, 30-m sprint)	Improvement in 1-RM half squat strength and performance in 30-m sprints	Increases muscle phosphocreatine levels, stabilizes muscle cell membranes, and regulates mitochondrial permeability to enhance energy metabolism	(13)
HMB	Fish, lean meat, citrus fruits, and dairy products	Adult men and women	3 g day^−1^ for 28 days	Strength (upper body, lean mass)	Increase in upper body strength and lean body mass	Inhibits protein degradation and promotes protein synthesis, improves mitochondrial biogenesis, and enhances fatty acid oxidation	(27)
		Elderly individuals	3 g day^−1^ for 84 days	Functional strength (SPPB score, relative muscle strength)	Improvement of cognitive function and increase in SPPB score and relative muscle strength		(16)
L-carnitine	Red meat, poultry, fish, and dairy products	Elderly individuals	2 g day^−1^ for 6 months	Functional endurance (6-min walk, mobility)	Cognitive function improvement, increased mobility, and enhanced 6-minute walking distance	Promotes fat oxidation, enhances antioxidant capacity, and reduces metabolite accumulation and the formation of fatigue-related neurotransmitters	(20)
		Adult men	2 g day^−1^ for 63 days	Strength (bench press, leg lift, peak power)	Increased bench press and leg lift weights, with improvements in average effectiveness and peak power		(19)
β-Alanine	Chicken, beef, fish, poultry, legumes, whole grains, and nuts	Elite cyclists	65 mg kg^−1^ day^−1^ for 28 days	Aerobic endurance (repetitions, fatigue index)	The increase in repetition frequency significantly reduces the fatigue index	Increases the content of carnosine, which plays a buffering and antioxidant role	(22)
		Elite climbers	4 g day^−1^ for 28 days	Aerobic/muscular endurance (climb frequency and duration)	The total number of climbs increased by 21%, the number of climbs per session increased by approximately 51%, and the duration extended by approximately 59%		(21)
Taurine	Meat, fish, dairy products, and eggs	Adult women	50 mg kg^−1^, single acute dose	Short-term endurance (power output test)	Increased terminal testing power output has led to a dramatic enhancement in short-term endurance performance	It may be related to promoting higher availability of calcium for cross-bridge formation and muscle contraction and may also be associated with antioxidant properties	(24)
		Adult men	50 mg kg^−1^, 2 hours pretest	Aerobic endurance in heat (time to exhaustion)	The time of exhaustion increased by 10%, and the rate of sweating at the end increased by 12.7%. During the last 10% of the exhausted time, the RPE and core temperature reduced in the later stages of exercise		(23)
NAC	Eggs, lean meat, poultry, seafood, dairy products, soybeans, garlic, onions, leeks, cauliflower, and broccoli	Adult men (IV administration)	125 mg kg^−1^ bolus (15 min) and 25 mg kg^−1^ h^−1^ infusion (20 min)	Aerobic endurance (time at 92% VO₂max)	Increased total glutathione levels and reduced glutathione levels in muscles; the endurance time at 92% of maximum oxygen uptake increased by 26.3%	Increases antioxidant capacity and reduces oxidative stress, including the protection of key ion channel proteins such as Na-K-ATPase	(26)
		Adult men (IV administration)	125 mg kg^−1^ bolus (15 min) and 25 mg kg^−1^ h^−1^ infusion (20 min)	Aerobic endurance (time at 92% VO₂peak)	The duration of exercise at 92% VO2peak intensity was extended by 23.8%		(25)

### 2.2 Proteins

The amino acids and their derivatives reviewed above represent the individual building blocks through which specific signaling targets such as mTORC1, the ubiquitin–proteasome system, and nitric oxide synthase are modulated. In whole foods, these building blocks are packaged together as intact proteins, which deliver a broader array of amino acids simultaneously and therefore engage multiple anabolic and anticatabolic pathways at once.

Proteins are high-molecular compounds composed of various amino acids. As mentioned previously, different functional factors in amino acids enhance physical strength or endurance, and since proteins contain various amino acids, most functional factors of protein types can simultaneously enhance both strength and endurance. Therefore, intact proteins confer performance benefits by engaging the same molecular targets as individual amino acids, including the mTORC1–S6K1–4E-BP1 axis for stimulating muscle protein synthesis and the insulin–IGF-1–PI3K–Akt cascade for promoting anabolism and glycogen storage, but they do so through a more sustained release profile that prolongs pathway activation (28). However, different proteins exhibit variations in effectiveness at the same dosage because of differences in amino acid types and proportion. Among proteins, the most representative functional factor is whey protein (WP), primarily composed of α-lactalbumin and β-lactoglobulin (29). The supplement with WP can significantly reduce muscle damage after exercise and markedly increase upper body strength (30). In addition, there have been improvements in maximum muscle strength, repetitions, and peak power, as well as an increase in fat oxidation rates and a reduction in carbohydrate oxidation (31). Animal proteins include fish protein (32, 33) and red meat protein that are similar to WP, both of which can significantly prolong the load-swimming time of mice (34), promote muscle synthesis (35), increase muscle strength, and reduce cellular damage (36), thereby significantly lowering postexercise muscle damage markers such as blood urea nitrogen and lactate levels.

Compared with animal protein, plant protein has a lower leucine content and lacks certain essential amino acids such as methionine and lysine, with a lower digestibility than animal protein (37). Typical examples include soy protein, pea protein, and rice protein. The supplement with soy protein after exercise can significantly improve grip strength, walking speed, and 6-min walking performance (38), reduce fatigue index (39), and significantly increase lean body mass, total muscle mass, muscle fiber cross-sectional area, and leg press one-repetition maximum (40), with effects on muscle damage and redox status comparable to that of WP (41). Rice protein has the highest biological value and protein score among cereal proteins, featuring a balanced amino acid composition with high content levels. Studies found that the intake of 48 g day^−1^ of rice protein after resistance exercise showed similar effects to whey protein in terms of muscle growth, bench press and leg press strength, and peak power increase (42). Pea protein is characterized by being free of lactose and cholesterol, and long-term supplementation can significantly increase bicep thickness and muscle strength; however, short-term supplementation does not show significant effects on recovery from exercise injuries (43). Although the leucine content in plant protein is lower, it contains more conditionally essential amino acids and non-essential amino acids and has unique advantages such as being suitable for vegetarians and those with lactose intolerance. The studies of proteins and bioactive peptides and their effects and mechanisms on enhancing physical strength and endurance are presented in Table 2.

**Table 2:** The studies of proteins and bioactive peptides and their effects and mechanisms on enhancing physical strength and endurance.

Active ingredients	Source	Subject/model	Dosage and duration	Exercise/assessment type	effect	Mechanism	References
WP	Milk	Female athletes	24 g day^−1^ for 28 days	Strength (1-RM bench press, agility)	Body fat percentage decreases and 1-RM bench press strength and agility increase	Rich in glutamine and leucine	(30)
		Adult men	25 g day^−1^ for 2 days	Anaerobic strength and power (1-RM, peak power)	Significant increases in maximum muscle strength, repetitions, and peak power		(31)
Grass carp protein versus peptide	Grass carp	Male ICR mice	1 or 5 mg g^−1^ day^−1^ for 28 days	Aerobic endurance (swimming time)	Swimming endurance time extension	Increases the reserves of liver and muscle glycogen and enhances the body's antioxidant capacity	(32)
Sea Bass essence	*Lates calcarifer* (Bloch)	Male ICR mice	1.04, 2.08, or 4.16 g kg^−1^ day^−1^ for 28 days	Aerobic endurance (swimming time to exhaustion)	The time to exhaustion in swimming is significantly prolonged	Rich in BCAAs	(33)
Red meat protein	Beef, veal, and lamb	Middle-aged men	0, 57, 113, or 170 g (single dose, 4 h pretraining)	Resistance exercise (protein synthesis assessment)	Gait speed and muscle density have both shown significant improvement	Red meat is rich in leucine, which can promote muscle synthesis	(34)
		Elderly women	160 g day^−1^ for 4 months	Strength (lean tissue mass, leg muscle quality)	Increased total lean tissue mass, leg lean tissue quality, and muscle strength		(36)
		Elderly adults (sex not specified)	80 g day^−1^ for 6 months	Functional strength (gait speed, muscle density)	Increased protein synthesis		(35)
Rice protein	Rice	Adult males	48 g day^−1^ for 8 weeks (postresistance exercise)	Strength (1-RM, peak power; muscle thickness)	Muscle thickness increased by 0.2 centimeters, strength in the 1-RM increased by 76.4 kilograms, and peak power rose from 638.4 watts to 753.9 watts	Rich in leucine	(42)
Pea protein	Pea	Adult men	0.9 g kg^−1^ postexercise (single dose)	Recovery/strength (muscle injury biomarkers)	The levels of biomarkers for muscle injury are significantly reduced	Rich in leucine	(43)
Soy protein	Soy	Stroke patients (sex not specified)	17.9 g day^−1^ for 8 weeks	Functional strength (grip, 8-ft walk, 6-min walk)	Significant improvements were observed in grip strength, 8-foot walking speed, and 6-minute walking performance	Soy protein is rich in lysine and isoleucine, which can promote muscle repair. In addition, soy protein contains isoflavones, which may inhibit the Akt and mTORC1 pathways, excessively suppressing inflammation and oxidative responses and reducing the bioavailability of IGF-1, which is detrimental to the enhancement of muscle strength	(38)
		Adult males	1.6 g kg^−1^ day^−1^ for 12 weeks	Strength (leg lean mass, muscle CSA, 1-RM leg press)	Significant improvements were observed in leg lean body mass, total muscle and muscle fiber cross-sectional area, and leg press one-repetition maximum (1-RM) levels		(40)
		Adult males (postsprint test)	21.5 g, single acute dose	Anaerobic endurance (fatigue index, sprint test)	The fatigue index has significantly decreased		(39)
		Male football players	1.5 g kg^−1^ (dose timing not specified)	Endurance (speed maintenance 48 h post-training)	The speed during the second high-speed run 48 hours after training is significantly reduced		(41)

### 2.3 Micronutrients

While proteins supply macromolecular substrates for muscle construction and repair, micronutrients serve as indispensable cofactors that regulate the enzymatic reactions underlying the same processes. Without adequate vitamins and minerals, the anabolic and energy-generating pathways activated by amino acids and proteins cannot function efficiently.

Vitamins and minerals are essential trace nutrients for the human body, playing a significant role in normal metabolism and overall health. Prolonged or intense muscle exercise often leads to the loss of some important trace elements, resulting in a decline in athletic performance. Ensuring that trace elements are not deficient is fundamental to maintaining physical strength and endurance. For example, in vitamins, vitamin B (VB) serves as a cofactor in the metabolism of fatty acids, carbohydrates, and amino acids (44). Athletes deficient in vitamin B1 (VB1), vitamin B2 (VB2), and vitamin B3 (VB3) experience a 16% reduction in aerobic exercise capacity and a 24% reduction in anaerobic exercise capacity, with short-term supplementation failing to restore these levels (45). In minerals, a deficiency in calcium may lead to exercise-induced stress fractures (46), and a lack of selenium can cause white muscle disease, resulting in muscle weakness and degeneration of cardiac and skeletal muscle, among other issues (47). Because of the lack of physiological mechanisms in the human body to independently synthesize these trace elements, an individual's reserves and nutritional status of trace elements entirely depend on dietary intake and the recycling processes within the body. Most studies have found that additional supplementation of trace nutrients is not beneficial for well-nourished athletes and may instead hinder the body's physiological adaptation to training. Therefore, most studies on functional factors related to physical endurance are based on models lacking these trace elements (48).

For simultaneously enhancing physical strength and endurance, several minerals with clear evidence of efficacy have been identified, such as selenium, iron, and magnesium. Selenium plays a crucial role in the human body's antiviral capacity, anticancer ability, and maintenance of normal muscle function. Research indicates that selenium supplementation can also increase muscle mass (49) and reduce oxidative stress (50). Supplementing with nano-selenium can reverse the reduction of selenium protein N in the muscles of aged mice (51) and enhance calcium ion release from the sarcoplasmic reticulum, muscle strength, and endurance (52). Iron is a functional component in oxygen transport (hemoglobin) and storage (myoglobin) and is essential for electron transfer reactions, gene regulation, and the processes of cell growth and differentiation (53). Iron supplementation significantly increases oxygen uptake, speed, and endurance time in individuals with anemia caused by iron deficiency during exercise (54). Magnesium plays an important role in maintaining muscle function and controlling inflammation. Research has found that magnesium supplementation in athletes who are not magnesium-deficient can still improve rebound jump ability and lower postexercise blood lactate levels (55). Acute magnesium supplementation has a positive effect on muscle strength and blood pressure, while chronic magnesium supplementation shows less pronounced effects (56). High doses of magnesium may positively influence functional performance and neuromuscular strength in young individuals, while adequate magnesium can enhance health functions and physical activity performance in the elderly (57). In addition, coenzyme Q10, as a vitamin-like substance, can enhance physical strength and endurance simultaneously. Animal studies have shown that supplementation with ubiquinol (the reduced form of coenzyme Q10) can significantly enhance the exercise performance of mice (58). Research on humans indicates that supplementation with coenzyme Q10 can significantly increase exercise output power (59), prolong time to exhaustion, enhance short-distance sprint performance (60), and reduce muscle damage (61).

Among micronutrients, only vitamin D (VD) has the effect of solely enhancing physical strength. VD mainly includes vitamin D2 (VD2) and vitamin D3 (VD3), which exert their effects through the expression of VD receptors in the skeletal muscle, controlling serum calcium levels to directly influence muscle contraction and reduce exercise-induced apoptosis of muscle cells, thereby enhancing muscle strength. For healthy athletes, studies have shown that long-term supplementation with low-dose VD (2,000 IU day^−1^) can significantly improve peak power and average power (62). Supplementation with moderate-dose VD (5,000 IU day^−1^) can increase strength and explosiveness as well as increase lean mass (63). For athletes deficient in VD, long-term supplementation with high doses (200,000 IU day^−1^) or acute supplementation with high doses (150,000 IU day^−1^) can significantly enhance jumping ability, muscle strength, and running speed (64).

At the pathway level, B-group vitamins function as coenzymes in the TCA cycle and the electron transport chain, directly supporting the ATP synthesis that underpins both strength and endurance. Vitamin D acts through the vitamin D receptor to regulate genes governing calcium handling and muscle protein turnover. Calcium and magnesium ions regulate actomyosin cross-bridge cycling and SERCA pump activity, thereby coupling micronutrient status to the same calcium homeostasis axis described in Section 4.3. Iron is a structural component of hemoglobin and myoglobin and is essential for the oxygen delivery pathway outlined in Section 4.1. The studies of micronutrients and their effects and mechanisms on enhancing physical strength and endurance are presented in Table 3.

**Table 3:** The studies of micronutrients and their effects and mechanisms on enhancing physical strength and endurance.

Active ingredients	Source	Subject/model	Dosage and duration	Exercise/assessment type	Effect	Mechanism	References
Selenium	Seafood, plant seeds, and animal organs	Aged male mice	5 mg kg^−1^ for 2 months	Strength and endurance (maximum contraction, fatigue resistance)	Increases muscle maximum contraction strength and fatigue resistance	By increasing the expression of selenoprotein N, enhancing antioxidant capacity, and protecting the function of calcium channels in the sarcoplasmic reticulum, the calcium release and contraction performance of the skeletal muscle have been improved	(52)
		Male mice	0.5 or 5 ppm day^−1^ nano-selenium for 14 days	Aerobic endurance (speed, running distance)	Increases the average speed, maximum speed, and total distance traveled		(51)
		Nellore ram lambs	0.019, 0.09, or 0.11 mg kg^−1^ day^−1^ for 120 days	Strength (muscle mass)	Muscle mass increase		(49)
		Growing beef heifers	0.5 kg day^−1^ for 105 days	Metabolic (mitochondrial gene expression; oxidative stress)	Increased mitochondrial gene expression capacity and reduced oxidative stress		(50)
Iron	Meat, nuts, legumes, leafy greens, and whole grains	Iron-deficient athletes (sex not specified)	200 mg day^−1^ for 12 weeks	Aerobic endurance (VO₂max; max speed, endurance time)	The maximum oxygen uptake, maximum speed, and endurance time have significantly increased	Iron is an important component of many enzymes and proteins, enhancing their conversion efficiency during physical activity	(54)
Magnesium	Dark green leafy vegetables, nuts, legumes, fruits, grains, and seafood	Male volleyball players	350 mg day^−1^ for 4 weeks	Anaerobic endurance (lactic acid, athletic performance)	Improving anaerobic metabolism, reducing lactic acid production, and enhancing athletic performance	By promoting the production of nitric oxide (NO) and other vasodilators in endothelial cells and inhibiting the release of vasoconstrictors, blood pressure is reduced; it affects the activity of calcium channels, regulating the contraction and relaxation of the vascular smooth muscle, and by influencing vascular resistance and vascular tension, it leads to a decrease in total peripheral resistance	(55)
		Adult volunteers (sex not specified)	300 mg day^−1^ for 1 week or 4 weeks	Strength (bench press performance)	Bench press performance significantly increased		(56)
Coenzyme Q10	Beef, chicken, pork, animal liver, fish, whole grains, nuts, seeds, spinach, cauliflower, and rapeseed	Male ICR mice	102.5, 205, or 615 mg kg^−1^ day^−1^ for 28 days	Strength and endurance (grip strength, loaded swimming time)	The grip strength of the forelimbs and the time spent swimming under load were both significantly increased	Increases oxygen supply by elevating the levels of 2,3-bisphosphoglycerate in red blood cells, improves mitochondrial biogenesis, enhances glycogen reserves, increases antioxidant capacity, and reduces metabolite accumulation	(58)
		Competitive swimmers (sex not specified)	300 mg day^−1^ for 12 days	Aerobic endurance and sprint (running time, short-distance swim time)	Exhausted running time is extended, while sprinting time for short-distance swimming is reduced		(60)
		Competitive swimmers (sex not specified)	300 mg day^−1^ for 14 days	Recovery (serum myocardial injury markers)	The level of serum myocardial injury markers is reduced		(61)
		Male cyclists	20 mg day^−1^ for 28 days	Aerobic endurance (time trial, average power output)	The average completion time has been reduced by 1.3%, while the average power output during the time trial has increased by 4.4%		(59)
VD	Fatty fish, egg yolks, dairy products, and fortified cereals	Adult men	2,000 IU day^−1^ for 12 weeks	Aerobic capacity and power (VO₂max, peak power, fatigue index)	Maximal oxygen uptake, peak power, and average power have significantly increased, while the fatigue index has significantly decreased	Enhances muscle function by regulating calcium ion levels and the gene expression of muscle cells, while reducing inflammatory responses through the modulation of immune system functions	(64)
		Competitive swimmers (sex not specified)	5,000 IU day^−1^ for 12 weeks	Strength (squat, vertical jump, bench press, lean mass)	There is a significant increase in lean body mass, and improvements are observed in performance during squats, vertical jumps, and bench presses		(63)
		Male football players (VD-deficient)	200,000 IU day^−1^ for 12 weeks	Strength and speed (jump, agility, running speed)	Jumping ability, agility, and running speed have significantly improved		(65)
		Judo athletes (VD-deficient)	150,000 IU day^−1^ for 8 days	Strength (muscle strength)	Significant increase in muscle strength		(62)

## 3 Natural extracts: animal, plant, and microbial sources

The paradigm for enhancing physical performance extends beyond essential nutrients to encompass natural extracts derived from traditional medicine. These extracts, obtained from animal, plant, and microbial sources, contain active compounds (including polysaccharides, saponins, and alkaloids) that are now identifiable through modern analytics. Their distinctive utility stems from a capacity to holistically modulate intricate physiological networks—spanning energy metabolism, oxidative stress, immune-inflammation, and neuroendocrine axes—via multitiered, synergistic mechanisms.

### 3.1 Animal-derived functional factors

Royal jelly is rich in fatty acids, primarily 10-hydroxy-2-decenoic acid and 10-hydroxydecanoic acid, which contribute to both enhanced strength and endurance. In forelimb grip strength and wire suspension tests, aged mice supplemented with royal jelly exhibited significant improvements in strength and endurance. This effect may be attributed to a marked reduction in the expression of MuRF1 and atrogin-1, thereby delaying age-dependent dysregulation of muscle mass through the modulation of genes involved in muscle regeneration and catabolism (66). In a previous study investigating allocentric spatial navigation ability, royal jelly supplementation was shown to promote more active search behavior in aged mice during the Morris water maze task, as evidenced by significantly increased swimming distance and speed, which reflects improved memory function (67). Considering the established evidence that royal jelly enhances muscle strength and endurance, a novel hypothesis can be proposed: this behavioral optimization may potentially represent a synergistic enhancement of central cognitive function and peripheral motor performance. Royal jelly may exert multitarget effects, simultaneously improving the brain's “command” capacity and the muscles' “executive” efficiency. In addition to the more typical royal jelly, similar effects have been found in studies of chicken essence and kefir for simultaneously enhancing physical endurance. MuRF1 and atrogin-1 are E3 ubiquitin ligases that act as key effectors of the ubiquitin–proteasome degradation pathway; their downregulation by royal jelly therefore converges on the same anticatabolic signaling axis modulated by HMB and leucine metabolites in the nutritional basis section. Kefir is a probiotic beverage fermented from milk, which not only increases glycogen reserves but also alters the composition of the gut microbiota, particularly by reducing the *Firmicutes*/*Bacteroides* ratio, and enhances nutrient availability through the short-chain fatty acids produced by lactic acid (68). Chicken essence is a liquid nutritional supplement made from cooking whole chickens. Chicken essence contains imidazole dipeptide, which is a class of important bioactive peptides, primarily including carnosine and anserine. Imidazole dipeptide can delay physically induced fatigue through its antioxidant activity, while effectively absorbing glucose for exercise energy by upregulating the translocation of GLUT4 protein, thereby reducing the accumulation of metabolites (69).

Supplementation with *Cervus elaphus* L. extract significantly enhanced swimming endurance and immune function in mice. The processed forms included extracts fermented with *Lactobacillus curvatus* strain HY7602 (70) or digested by YC-1101 (71). This effect may be attributed to the ability of deer antlers to downregulate the mRNA expression of IL-1β, IL-6, and TNF-α in the muscles, thereby reducing the expression of proinflammatory cytokines. In addition, it may activate the Nrf2–Keap1 pathway to increase antioxidant enzyme levels, thus mitigating oxidative stress. An example similar to this is the decapeptide CMS001, which is a peptide component isolated and extracted from bovine spleen. It enhances the body's antioxidant capacity by reducing the accumulation of metabolites produced by oxidative stress (such as MDA) and increases glycogen reserves during exercise, significantly extending the swimming time to exhaustion in mice (72). The studies of animal-derived functional factors regarding their effects and mechanisms for enhancing physical strength and endurance are summarized in Table 4.

**Table 4:** The studies of animal-derived functional factors regarding their effects and mechanisms for enhancing physical strength and endurance.

Active ingredients	Source	Subject/model	Dosage and duration	Exercise/assessment type	Effect	Mechanism	References
Royal jelly	Bee	Aged heterogeneous male mice (6–36 months)	0.05% or 0.5% royal jelly in daily feed from 6 to 36 months of age	Strength and endurance (grip strength, wire hanging, pull-ups, rotarod)	Improved muscle atrophy caused by aging, significantly enhancing performance in grip strength, wire hanging, pull-ups, and rotation bar tests	Enhances antioxidant capacity and reduces muscle atrophy	(66)
Kefir	Milk	Male ICR mice	2.15, 4.31, or 10.76 g kg^−1^ day^−1^ for 28 days	Strength and endurance (grip strength, swimming time to exhaustion)	The time to exhaustion during swimming is significantly extended, and forelimb grip strength increases	Regulates the intestinal microbiota, increases glycogen reserves, and reduces the accumulation of metabolites	(68)
Chicken essence	Hen	Male ICR mice	845, 1,690, or 4,225 mg kg^−1^ day^−1^ for 4 weeks	Strength and endurance (grip strength, swimming time)	Enhanced grip strength and increased swimming endurance time	Enhances antioxidant capacity, increases glycogen reserves, and reduces the accumulation of metabolites	(67)
*Cervus elaphus* L.	Deer	Male ICR mice	120 mg kg^−1^ day^−1^ for 120 days (YC-1101 digested)	Aerobic endurance (swimming time to exhaustion)	Exhaustion significantly extends swimming time	Enhances antioxidant capacity and reduces the production of proinflammatory factors by strengthening immunity	(71)
		Male ICR mice	50, 62.5, or 100 mg kg^−1^ day^−1^ for 21 days (HY7602 fermented)	Aerobic endurance (swimming time to exhaustion)	Exhaustion significantly extends swimming time	Enhances antioxidant capacity, reduces oxidative stress, and decreases the accumulation of metabolites	(70)
The decapeptide CMS001	Pig spleen	Male Kunming mice	5 or 20 μg kg^−1^ day^−1^ for 30 days	Aerobic endurance (swimming time to exhaustion)	The time to exhaustion in swimming is significantly prolonged	Enhances antioxidant capacity, increases glycogen reserves, and reduces the accumulation of metabolites	(72)

### 3.2 Microbial sources’ functional factors

Compared with the animal-derived functional factors reviewed above, which act largely through protein-based bioactive compounds that modulate anabolic signaling and reduce muscle catabolism, microbial-derived functional factors share some immune and antioxidant properties but are distinguished by a primary focus on enhancing endurance rather than strength, acting through mechanisms that are detailed in this subsection.

Most functional factors derived from microorganisms primarily serve to enhance endurance, with typical representatives being *Cordyceps sinensis* (CS), *Hericium erinaceus* (HE), and *Ganoderma lucidum* (CL). The main active components of CS include nucleosides, polysaccharides, alkaloids, polyamines, organic acids, and vitamins. Supplementation with CS mycelium can significantly extend the exhaustion swimming time of mice (73). The mechanism may involve the upregulation of adenosine 5'-monophosphate-activated protein kinase (AMPK), peroxisome proliferator-activated receptor gamma coactivator 1-alpha (PGC1-α), and PPAR expression, as well as the upregulation of MCT1 and GLUT4 expression to improve metabolic transport (74). In addition, it may reduce oxidative stress by activating the Nrf2 antioxidant pathway (75, 76). The CL is rich in polysaccharides, peptides, triterpenes, amino acids, nucleosides, enzymes, organic germanium, sterols, alkaloids, furan derivatives, and various trace elements. CL polysaccharides can significantly extend the swimming time of mice, potentially by inhibiting the levels of inflammatory factors such as TNF-α and IL-6, thereby enhancing immunity and reducing oxidative stress through increased SOD activity (77). Polysaccharides of HE and *Tuber melanosporum* (TM) enhances endurance by increasing antioxidant capacity, boosting glycogen reserves, and regulating hormone levels in the body (78, 79). At the molecular level, the endurance-enhancing effects of these microbial extracts are mediated by two key signaling axes. The AMPK/PGC1-α pathway promotes mitochondrial biogenesis and fatty acid oxidation and elevates the proportion of oxidative muscle fibers. Inhibition of the NF-κB inflammatory pathway reduces exercise-induced cytokine release and thus accelerates physical recovery and prolongs time to exhaustion. The studies of the functional factors of microbial sources regarding their effects and mechanisms for enhancing physical strength and endurance are summarized in Table 5.

**Table 5:** The studies of microbial sources functional factors regarding their effects and mechanisms for enhancing physical strength and endurance.

Active ingredients	Source	Subject/model	Dosage and duration	Exercise/assessment type	Effect	Mechanism	References
CL	*Ganoderma lucidum* (Curtis) P. Karst	Male ICR mice	50, 100, or 200 mg kg^−1^ day^−1^ for 21 days	Aerobic endurance (swimming time to exhaustion)	The time to exhaustion during swimming is significantly prolonged	Enhances antioxidant capacity and strengthens immunity	(77)
CS	*Cordyceps sinensis* (Berk.) Sacc	Male Sprague-Dawley rats	200 mg kg^−1^ day^−1^ for 15 days	Aerobic endurance (swimming time)	Exercise endurance has increased by 1.79 times	Increases glycogen reserves and enhances antioxidant capacity	(75)
		Male ICR mice	45.5, 91, or 182 mg kg^−1^ day^−1^ for 30 days	Aerobic endurance (swimming time to exhaustion)	The time to exhaustion in swimming is significantly prolonged and is positively correlated with dosage	Reduces oxidative stress by enhancing antioxidant capacity	(74)
		Male Sprague-Dawley rats	40, 80, or 160 mg kg^−1^ day^−1^ for 28 days	Aerobic endurance (swimming time to exhaustion)	The time to exhaustion in swimming is significantly extended	Increases antioxidant capacity, reduces metabolite accumulation, and enhances glycogen reserves	(73)
		Male Sprague-Dawley rats	100, 200, or 400 mg kg^−1^ day^−1^ for 28 days	Aerobic endurance (swimming time to exhaustion)	The time to exhaustion in swimming is significantly extended	Increases antioxidant capacity, reduces metabolite accumulation, and enhances glycogen reserves	(76)
TM	*Tuber melanosporum* Vittad	Male ICR mice	25, 50, or 100 mg kg^−1^ day^−1^ for 21 days	Aerobic endurance (swimming time, rotarod, running time)	The time for swimming to exhaustion increased by 51%–58%, the time for spinning increased by 67%–117%, and the running time increased by 40%–78%	Increases antioxidant capacity, enhances glycogen reserves, and regulates hormone levels in the body	(78)
HE	*Hericium erinaceus* (Bull.) Pers.	Male Sprague-Dawley rats	50, 100, or 200 mg kg^−1^ day^−1^ for 28 days	Aerobic endurance (swimming time to exhaustion)	The time to exhaustion in swimming is significantly extended	Reduces metabolite accumulation and enhances antioxidant capacity	(79)

### 3.3 Plant-derived functional factors

Both the animal- and microbial-derived functional factors reviewed above act through relatively concentrated bioactive compounds such as peptides, polysaccharides, and nucleosides. Plant-derived functional factors represent the most chemically diverse category, encompassing complex extracts, polysaccharides, polyphenols, terpenes, and other compounds that collectively engage a broader range of signaling targets. The following subsections review these factors by chemical class.

#### 3.3.1 Plant complex extracts

The components of the effects of plant complex extracts mainly focus on enhancing endurance, specifically manifested as an extension of the time until exhaustion during exercise. Most plant complex extracts possess antioxidant capabilities. For instance, supplementation with the alcoholic extract of *Polygonatum alte-lobatum* Hayata rhizomes (containing polyphenols, flavonoids, and polysaccharides) can increase superoxide dismutase activity, enhance antioxidant capacity, and prolong the exhaustion running time of mice by 1.43–1.62 times (80). *Euterpe oleracea* Mart. seed extract (composed of proanthocyanidins, catechin, and epicatechin) (81) and *Lepidium meyenii* Walp. extract (composed of N-benzylpalmitamide, N-benzyloleamide, and N-benzyl-linoleamide) (82, 83) can significantly prolong the swimming time to exhaustion in mice by enhancing mitochondrial biogenesis and protecting mitochondria. The extract of *Lycium barbarum* L. (the main component of water-soluble *L. barbarum* L. polysaccharide) upregulates the expression of ERRγ by activating the PKA–CREB signaling pathway, increasing the proportion of oxidative muscle fibers type IIa, resulting in an approximately 40% increase in running distance in mice (84). The aqueous extract (component of rhamnose, glucosinolates, and isothiocyanates) (85) and *Ludwigia octovalvis* (Jacq.) Raven extract (a component of polyphenols, flavonoids, and β-sitosterol) (86) can significantly prolong the swimming time to exhaustion in mice by increasing glycogen reserves. The hydroalcoholic extract of *Aegle marmelos* fruit (a component of chlorogenic acid, tannic acid, ferulic acid, gallic acid, and quercetin) can increase the swimming time to exhaustion in mice by 47.5% through the reduction of metabolite accumulation (87). *Astragalus membranaceus* (Fisch.) Bunge radix extract (a component of astragaloside IV and calycosin-7-O-b-D-glucoside) promotes fat metabolism and reduces glycogen utilization by activating the AMPK pathway, significantly extending the time to exhaustion in swimming. In addition, it can modulate gut microbiota metabolism through the reduction of trimethylamine levels, acting in concert with the aforementioned effects (88).

Research on the effects of plant composite extracts that solely enhance physical performance is limited. There is clear evidence for powdered montmorency tart cherry, beetroot concentrate, and golden root extract. Studies indicate that athletes supplementing with powdered montmorency tart cherry improve their half-marathon times by an average of 13%, likely due to its immune-boosting properties (89). Beetroot concentrate (composed of betalains) can significantly enhance the performance of cyclists, with mechanisms that may include reduced oxidative stress and increased blood flow (90). Golden root extract enhances anaerobic performance by increasing ATP-PC reserves within muscles, promoting the synthesis or resynthesis of ATP, and facilitating the recruitment of motor units through neural regulation (91).

#### 3.3.2 Polysaccharides

Polysaccharides are large molecules composed of various monosaccharides and have long been regarded as a novel, natural, and effective substance for combating fatigue. Among the functional factors derived from plant-based polysaccharides, the primary effect is to enhance endurance. In addition to the generally recognized antioxidant capabilities of plant-derived functional factors, some functional factors enhance endurance through various mechanisms.

First, from the perspective of energy metabolism, *Malpighia emarginata* DC. polysaccharides can enhance energy metabolism by increasing mitochondrial biogenesis (92). To increase energy storage, *Dendrobium officinale* polysaccharides enhance fat mobilization and utilization, while reducing glycogen and protein consumption (93). Polysaccharides from *Achyranthes bidentata* (94) and *Millettiae speciosae* Champ (92) have extended exercise duration by increasing glycogen reserves. Second, from an immunological perspective, the polysaccharide of *Pseudostellaria heterophylla* enhances immunity and prolongs endurance by increasing the number of T lymphocytes in the thymus and spleen, as well as raising the CD4^+^/CD8^+^ ratio (95). Finally, the research found that not only can the animal-derived functional factor kefir enhance physical strength by adjusting the gut microbiota, but plant-derived polysaccharide functional factors such as konjac oligosaccharide polysaccharides can also alter the gut microbial structure in mice. This is achieved by selectively increasing the abundance of several beneficial bacteria such as *Lactobacillus gasseri* and *Bifidobacterium pseudolongum*, promoting the growth of cecal probiotics and the production of short-chain fatty acids, thereby significantly extending exercise endurance (96). In addition, most functional factors have the effect of reducing the accumulation of metabolites, which is mainly related to enhancing immunity and antioxidant capacity, and is an important mechanism for improving endurance (97).

*A. membranaceus* (Fisch.) Bunge polysaccharides and *Polygonatum sibiricum* polysaccharide can simultaneously enhance physical strength and endurance, specifically manifested as a significant increase in exhaustion swimming time and a notable increase in grip strength. A study found that excessive exercise may trigger mitochondrial autophagy, and supplementation with *A. membranaceus* (Fisch.) Bunge polysaccharides can promote the expression of *MFN1* and *MFN2* genes in mitochondria, restoring mitochondrial dysfunction, thereby enhancing both physical strength and endurance (98). In a mouse aging model induced by D-galactose, *Polygonatum sibiricum* Redouté polysaccharide regulates the function of mitochondria-associated membranes (MAMs) by downregulating the expression of key proteins such as IP3R, GRP75, and VDAC1, as well as decreasing the expression of the mitochondrial calcium uniporter (MCU), maintaining intracellular calcium homeostasis (99). At the molecular level, plant-derived polysaccharides commonly activate the AMPK/SIRT1/PGC1-α axis to promote mitochondrial biogenesis and glycogen synthesis, while also engaging Toll-like receptor signaling pathways to enhance immune function and reduce exercise-induced inflammation.

#### 3.3.3 Polyphenols and flavonoids

Polyphenols are a class of compounds with significant antioxidant activity, specifically divided into flavonoids and non-flavonoids. Among flavonoid polyphenols, quercetin, tangeretin, and dihydromyricetin (DHM) are noted, with effects primarily focused on enhancing endurance (100). In non-flavonoid polyphenols, there is clear evidence that epigallocatechin gallate (EGCG), curcumin, and anthocyanins promote physical endurance, although the effects are different. Research indicates that supplementation with quercetin, hesperidin (101), and DHM (102) enhances endurance in mice primarily by promoting mitochondrial biogenesis. However, the specific mechanisms are slightly different. Quercetin and tangeretin primarily enhance mitochondrial biogenesis by activating the AMPK/PGC1-α/NRF1/transcription factor A, mitochondrial (TFAM) signaling pathway, which increases mitochondrial DNA replication and gene transcription. In addition, quercetin may exert stimulant-like effects by antagonizing adenosine A1 receptors. In contrast, DHM mainly regulates the dynamic balance of mitochondria by upregulating genes related to mitochondrial fusion (*MFN1*, *MFN2*) and downregulating genes associated with mitochondrial fission, such as dynamin-related protein 1 (*DRP1*) and Fission 1 (*FIS1*), thereby promoting the structural remodeling of mitochondria.

Non-flavonoid functional factors exhibit slight differences in their effects. Among them, EGCG and hawthorn berry polyphenols can enhance exercise endurance, while anthocyanins and epicatechin can improve both physical endurance and strength, and curcumin is more focused on enhancing physical strength. Research indicates that continuous supplementation with EGCG for 28 days can extend the swimming time to exhaustion in mice under load, significantly increasing muscle glycogen levels and antioxidant capacity (103). Its mechanism of action centers on increasing muscle glycogen content to prolong endurance. The main active components of hawthorn polyphenols are procyanidin B2, epicatechin, and chlorogenic acid. Supplementation can effectively extend the swimming time to exhaustion in fatigued mice (104); the related mechanisms include inhibiting the expression of proinflammatory factors such as TNF-α and IL-1β, regulating the PI3K/Akt/GSK-3β pathway to improve mitochondrial dysfunction and cellular metabolism, while suppressing the NF-κB inflammatory pathway. In addition, the mechanism of hawthorn polyphenols may be related to the regulation of gut microbiota (104). In terms of simultaneously improving endurance and physical strength, a continuous 7-day supplementation with anthocyanins, which are extracted from New Zealand blackcurrant, can significantly enhance the performance of male cyclists in competitions, reducing completion time by 0.82% (105). Anthocyanins can enhance both physical endurance and strength by reducing oxidative stress and preventing interference with the activity of the sodium–potassium pump. Clinical studies have confirmed that daily oral supplementation with epicatechin at 1 mg·kg^−1^·day^−1^ for 7 days can significantly improve handgrip strength in subjects (106). The core mechanism of action of epicatechin is the inhibition of myostatin, which belongs to the transforming growth factor-β (TGF-β) superfamily and acts as an endogenous negative regulator of skeletal muscle mass. Epicatechin downregulates myostatin gene expression and elevates the follistatin to myostatin ratio, thereby relieving the inhibitory effect of myostatin on myogenesis, activating the PI3K/Akt/mTOR pathway, and subsequently promoting muscle protein synthesis and satellite cell differentiation (107). In addition, epicatechin can increase skeletal muscle mitochondrial density by upregulating PGC-1α and AMPK expression and enhancing mitochondrial cristae abundance; animal experiments have confirmed that it can extend the maximum running distance and enhance muscle fatigue resistance in mice (108), and clinical studies have also observed significant increases in mitochondrial biogenesis–related protein levels and improvements in aerobic metabolic efficiency in patients with Becker muscular dystrophy (109). In terms of enhancing physical strength, daily supplementation with 200 mg of curcumin can increase the maximum exercise speed of subjects on a treadmill (110). Curcumin, the principal curcuminoid of turmeric (*Curcuma longa* L.), has been extensively used in ayurvedic and Chinese medicine for its anti-inflammatory and putative “blood-activating” properties, which align with its modernly discovered effects on muscle recovery and strength. Curcumin mainly enhances physical strength by boosting immune functions. It downregulates the expression of several proinflammatory cytokines involved in protein hydrolysis and muscle inflammation and inhibits the nuclear factor kappa-light-chain-enhancer of the activated B cell signaling pathway. This substance exerts its effects by inhibiting the nuclear factor kappa-light-chain-enhancer of the activated B cell (NF-κB) transcription factor to suppress proinflammatory cytokine production. It also activates the Nrf2/HO-1 antioxidant pathway and the AMPK/SIRT1/PGC1-α axis to enhance mitochondrial biogenesis and metabolic efficiency during sustained exercise. Across polyphenol subclasses, a recurring mechanistic theme is the concurrent suppression of NF-κB-mediated inflammatory signaling and activation of the PI3K/Akt/GSK-3β pathway, which together reduce muscle catabolism, improve glycogen metabolism, and maintain mitochondrial membrane integrity during exercise. The studies of polyphenols and flavonoids factors regarding their effects and mechanisms for enhancing physical strength and endurance are summarized in Table 6.

**Table 6:** The studies of plant-derived functional factors regarding their effects and mechanisms for enhancing physical strength and endurance.

Active ingredients	Source	Subject/model	Dosage and duration	Exercise/assessment type	Effect	Mechanism	References
*Euterpe oleracea* Mart. seed extracts	*Euterpe oleracea* Mart	Male Wistar rats	200 mg kg^−1^ day^−1^ for 4 weeks	Aerobic endurance (exercise time, distance)	Better exercise time and distance	Enhances antioxidant capacity and increases mitochondrial biogenesis	(81)
*Lycium barbarum* mimics extracts	*Lycium barbarum* mimics	Male C57BL/6 mice	2.5 g kg^−1^ day^−1^ for 120 days	Aerobic endurance (running distance)	Significantly extends the average running distance of mice	Increases the proportion of type I muscle fibers through the PKA–CREB–ERRγ pathway, enhancing aerobic metabolism	(84)
The Aqueous Extract of *Moringa oleifera*	*Moringa oleifera* Lam.	Male Sprague-Dawley rats	100, 200, or 400 mg kg^−1^ for 28 days	Aerobic endurance (swimming time to exhaustion)	The time until exhaustion in swimming is significantly extended	Reduces the accumulation of metabolites, increases glycogen reserves, and enhances antioxidant capacity	(85)
Kai Xin San (KXS)	*Panax ginseng* C.A. Mey., *Poria cocos* (Schw.) Wolf, *Polygala tenuifolia* Willd., *Acorus tatarinowii* Schott	Male Sprague-Dawley rats	175, 350, or 700 mg kg^−1^ for 28 days	Aerobic endurance (running time to exhaustion)	The time for running to exhaustion is significantly extended	Reduces the accumulation of metabolites	(123)
Hydroalcoholic extract of *Aegle marmelos* fruit	*Aegle marmelos* (L.) Corrêa	Male Swiss mice	100, 200, or 400 mg kg^−1^ day^−1^; tests on days 4, 7, 10, and 13	Aerobic endurance (swimming time to exhaustion)	The time until exhaustion in swimming is significantly extended	Reduces the accumulation of metabolites, promotes fat metabolism, and upregulates the skeletal muscle metabolic regulator GLUT4	(87)
Astragali Radix extracts	*Astragalus membranaceus* (Fisch.) Bunge	Male Sprague-Dawley rats	1, 3, or 6 g kg^−1^ day^−1^ for 21 days	Aerobic endurance (swimming time to exhaustion)	The time until exhaustion in swimming is significantly extended	Promotes lipolysis, increases citrate levels to regulate energy metabolism, reduces trimethylamine levels to modulate gut microbiota metabolism, and decreases O-acetylated glycoprotein levels to alleviate inflammatory responses	(88)
*Ludwigia octovalvis* (Jacq.) Raven extract	*Ludwigia octovalvis* (Jacq.) Raven	Male ICR mice	61.5 or 307.5 mg kg^−1^ day^−1^ for 28 days	Aerobic endurance (swimming time to exhaustion)	The time until exhaustion in swimming is significantly extended	Increases glycogen reserves and enhances antioxidant capacity	(86)
Powdered Montmorency tart cherry	*Prunus cerasus* L. var. acida (Moench) Pers.	Male and female trained athletes	480 mg day^−1^ for 28 days	Aerobic endurance (race completion time, metabolite/inflammatory markers)	Reduction in the completion time of the competition, in metabolite accumulation, in inflammatory markers, and in the perception of soreness	Enhances immunity and antioxidant capacity through NF-κB-mediated mechanisms	(89)
Beetroot concentrate	*Beta vulgaris* L. subsp. vulgaris var. rapa Dumort	Male cyclists	100 mg day^−1^ for 7 days	Aerobic endurance (power output, exercise efficiency, blood flow)	Power output and exercise efficiency increase, blood flow increases	No increase in plasma NO*_x_* levels was observed, but blood flow may be improved through other unclear mechanisms	(90)
Alcoholic extract of *Polygonatum Alte-lobatum* Hayata rhizomes	*Polygonatum Alte-lobatum* Hayata rhizomes	Male Sprague-Dawley rats	75, 150, or 375 mg kg^−1^ day^−1^ for 56 days	Aerobic endurance (running time to exhaustion)	The time for exhaustive running is extended by 1.43 to 1.62 times	Enhances antioxidant capacity and reduces metabolite accumulation	(80)
*Macamides* extracts	*Lepidium meyenii* Walp.	Male ICR mice	12 or 40 mg kg^−1^ day^−1^ for 21 days	Aerobic endurance (swimming time to exhaustion)	The time until exhaustion in swimming is significantly extended	Increased fatty acid metabolism, reduced accumulation of metabolites, increased activity of antioxidant enzymes, and decreased oxidative stress	(82)
		Male Sprague-Dawley rats	4, 5.3, or 8 g kg^−1^ day^−1^ for 15 days	Aerobic endurance (swimming time, submersion count)	The total swimming time increased by a maximum of 94.47%, while the number of times submerged decreased by a maximum of 57.96%	Enhances antioxidant capacity, increases glycogen reserves, and protects mitochondria	(83)
Golden root extract	*Rhodiola rosea* L.	Adult women	1,500 mg day^−1^ for 3 days	Anaerobic power (average, peak, and total power)	The average power, peak power, and total power of anaerobic exercise have significantly increased	Increases phosphocreatine reserves; enhances central nervous system excitation	(91)
*Dendrobium officinale* polysaccharides	*Dendrobium officinale* Kimura et Migo	Male Sprague-Dawley rats	50 mg kg^−1^ day^−1^ for 30 days	Aerobic endurance (swimming time to exhaustion)	The swimming time of the supplemented mice was significantly extended	Increases fat metabolism, enhances antioxidant capacity, strengthens immunity, and reduces metabolite accumulation	(93)
*Achyranthes bidentata* polysaccharides	*Achyranthes bidentata* Blume	Male Sprague-Dawley rats	50, 100, or 200 mg kg^−1^ day^−1^ for 28 days	Aerobic endurance (swimming time to exhaustion)	The supplementary group's swimming time was significantly extended and showed a positive correlation with dosage	Increases liver glycogen and muscle glycogen content and reduces blood lactate and blood urea nitrogen levels	(94)
*Millettiae speciosae* Champ. polysaccharides	*Millettiae speciosae* Champ.	Male ICR mice	200, 400, or 800 mg kg^−1^ day^−1^ for 28 days	Aerobic endurance (swimming time to exhaustion)	The supplementary group's swimming time is significantly extended and is positively correlated with dosage	Increases liver glycogen and muscle glycogen content and reduces blood lactate and blood urea nitrogen levels	(92)
*Portulaca oleracea* L. polysaccharides	*Portulaca oleracea* L.	Male ICR mice	100, 200, or 400 mg kg^−1^ day^−1^ for 28 days	Aerobic endurance (swimming time to exhaustion)	The supplementary group's swimming time is significantly prolonged and is positively correlated with dosage	Reduces oxidative stress	(124)
*Pseudostellaria heterophylla* polysaccharides	*Pseudostellaria heterophylla* (Miq.) Pax ex Pax et Hoffm.	Male ICR mice	100, 200, or 400 mg kg^−1^ day^−1^; tests on days 1, 4, 7, 10, and 13	Aerobic endurance (swimming time to exhaustion)	The swimming time of the supplemented group was significantly prolonged, showing a positive correlation with dosage and supplementation duration	Enhances immunity	(95)
Maca Polysaccharides	*Lepidium meyenii* Walp.	Male ICR mice	400 mg kg^−1^ day^−1^ for 30 days	Aerobic endurance (swimming time, fatigue metabolites)	Supplemental group swimming time is significantly extended, and the accumulation of fatigue metabolites is reduced	Reduces the accumulation of metabolites	(125)
*Polygonatum sibiricum* Redouté polysaccharides	*Polygonatum sibiricum* Redouté	D-galactose-induced aging male mice	50 or 100 mg L^−1^ (dose in drinking water)	Strength and endurance (grip strength; wire hanging; wheel test)	Significant improvement in grip strength, hanging time, and wheel test performance in aging models	Maintains mitochondrial function, enhances antioxidant capacity, and reduces oxidative stress	(99)
*Malpighia emarginata* DC. polysaccharides	*Malpighia emarginata* DC.	Male ICR mice	50, 100, or 200 mg kg^−1^ day^−1^ for 28 days	Aerobic endurance (swimming time to exhaustion)	The time to exhaustion in swimming has significantly increased	Enhances antioxidant capacity and promotes mitochondrial biogenesis	(126)
Astragalus polysaccharides	*Astragalus membranaceus* (Fisch.) Bunge	Male ICR mice	100 mg kg^−1^ day^−1^ for 28 days	Strength and endurance (swimming time, grip strength)	The time spent swimming to exhaustion has significantly increased, as also grip strength	Increases mitochondrial biogenesis, enhances antioxidant capacity, and reduces oxidative stress	(98)
*Dimocarpus longan* Lour. polysaccharides	*Dimocarpus longan* Lour	Male ICR mice	50, 100, 200, or 400 mg kg^−1^ day^−1^ for 30 days	Aerobic endurance (swimming time to exhaustion)	The time for exhaustive swimming has significantly increased	Increases glycogen reserves and reduces metabolite accumulation	(97)
Konjac oligosaccharide polysaccharides	*Amorphophallus konjac* C. Koch	Male C57BL/6 mice	0.5, 1, or 2 g kg^−1^ day^−1^ for 35 days	Aerobic endurance (swimming time to exhaustion)	The time for exhaustive swimming has significantly increased	Through the growth of probiotics, the production of short-chain fatty acids, the increase of glycogen reserves, the reduction of metabolite accumulation, and the enhancement of antioxidant capacity	(96)
Quercetin	Onion, Apple, Tea leaves, Grape, Blueberry, Cherry, Broccoli, and Chili pepper	Male C57BL/6 mice	12.5 or 25 mg kg^−1^ day^−1^ for 7 days	Aerobic endurance (running wheel, running time, peak speed)	Maximum exercise endurance increased by 36%, with improvements observed in voluntary running wheel experiment in terms of distance, time, and peak speed	Promotes mitochondrial biogenesis by influencing nervous system function and regulating cellular signaling	(127)
Tangeretin	Citrus fruits, Lemon, Orange, Tangerine peel, and Grapefruit	Male ICR mice	25, 50, or 100 mg kg^−1^ day^−1^ for 28 days	Endurance (suspension time, physical exhaustion time)	The suspension time and the time of physical exhaustion are significantly extended	Promotes mitochondrial biogenesis	(101)
DHM	*Ampelopsis grossedentata* (Hand. Mazz.) W.T. Wang	Male Sprague-Dawley rats	50, 75, or 100 mg kg^−1^ day^−1^ for 7 days	Aerobic endurance (running time to exhaustion)	The time to exhaustion during running is significantly extended, reducing the accumulation of metabolites	By maintaining mitochondrial biogenesis and dynamics, the accumulation of metabolites is reduced	(102)
EGCG	*Camellia sinensis* (L.) Kuntze	Male ICR mice	50, 100, or 200 mg kg^−1^ day^−1^ for 28 days	Aerobic endurance (swimming time to exhaustion)	Significant extension of time until exhaustion during swimming	Increases glycogen content in muscles, reduces the accumulation of metabolites, and enhances antioxidant capacity	(103)
Epicatechin	*Theobroma cacao* L.	Healthy adults	1 mg·kg^−1^·day^−1^ for 7 days	Strength (grip strength)	Significantly improved handgrip strength; elevated follistatin/myostatin ratio	Inhibits myostatin, activates PI3 K/Akt/mTOR, and increases mitochondrial density	(106)
Curcumin	*Curcuma longa* L.	Adult men	200 mg day^−1^ for 4 days	Strength (maximum movement speed on treadmill)	Increase in maximum movement speed	Increases immunity and reduces the occurrence of inflammation	(110)
Anthocyanin	*Ribes nigrum* L.	Male cyclists	105 mg day^−1^ for 7 days	Aerobic endurance (cycling competition time)	The total time of the competition has been reduced by 0.82%	Reduces oxidative stress	(105)
Hawthorn berry polyphenols	*Crataegus pinnatifida* Bunge	Male ICR mice	200 mg kg^−1^ day^−1^ for 28 days	Aerobic endurance (swimming time to exhaustion)	Exhaustive swimming time is significantly prolonged, improving the imbalance of gut microbiota in mice	By enhancing mitochondrial function and cellular metabolism, inhibiting the NF-κB inflammatory pathway, and regulating gut microbiota, antioxidant capacity is improved	(104)
Ginsenoside Rg1	*Panax ginseng* C. A. Meyer.	Male Sprague-Dawley rats	50 mg kg^−1^ day^−1^ for 14 days	Aerobic endurance (spontaneous activity distance, baton-passing time)	The distance of spontaneous activities and the baton-passing time have been significantly extended	Regulating the expression of EGFR in the prefrontal cortex by modulating key metabolic markers such as taurine and mannose-6-phosphate	(112)
		Male Sprague-Dawley rats (fatigue model)	5 mg kg^−1^ day^−1^ for 7 days (surgery on day 3)	Functional endurance (walking distance, standing frequency)	The walking distance and the number of times standing significantly increased, while the rest time significantly decreased	Enhances antioxidant capacity	(111)
Eleutheroside	*Eleutherococcus senticosus* (Rupr. & Maxim.) Maxim.	Adult men	800 mg day^−1^ for 8 weeks	Aerobic endurance (endurance time, maximum heart rate)	Endurance time significantly increased by 23%, and the maximum heart rate rose by 4% during exhaustive exercise	Promotes fat metabolism, regulates insulin secretion and glucose utilization, lowers blood sugar, enhances cardiovascular function, and improves maximum heart rate and oxygen uptake	(113)
Ursolic Acid	*Arctostaphylos uva-ursi* (L.) Spreng.	Male ICR mice	80 or 240 mg kg^−1^ day^−1^ for 3 weeks	Aerobic endurance (suspended and weighted swimming time)	The time for suspended and weighted swimming has been significantly extended	Promotes mitochondrial biosynthesis	(114)
Caffeine	*Coffea arabica* L.	Male recreational runners	3 mg kg^−1^, single acute dose	Aerobic endurance and cognitive (speed, attention, pain sensation)	The speed increased by 1.1%, and there was a 15.6% improvement in attention and reaction time. In psychological tests, the favorability rating improved by 15.7%, the perception of stress decreased by 17.6%, and the sensation of pain was reduced by 11.3%	By antagonizing adenosine receptors (A1 and A2a) in the central nervous system, it may also improve psychological state and cognitive function by indirectly promoting the secretion of dopamine	(115)
CAP	*Capsicum annuum* L.	Male ICR mice	205, 410, or 1,025 mg kg^−1^ for 28 days	Strength and endurance (grip strength, swimming time to exhaustion)	Significant improvement in grip strength and swimming exhaustion time	By activating the TRPV1 receptor, it promotes mitochondrial biosynthesis and ATP production, increases liver glycogen reserves, reduces the accumulation of metabolites, and enhances antioxidant capacity	(116)
Lycopene	*Solanum lycopersicum* L.	Male C57BL/6 mice	100,000 mg kg^−1^ continuously for 42 days	Aerobic endurance (swimming time to exhaustion)	Exhaustive swimming time significantly enhances	Increases the proportion of Type I muscle fibers through the AMPK/SIRT1/PGC1-α pathway	(117)
Octacosanol	*Saccharum officinarum* L., *Oryza sativa* L., *Triticum aestivum* L.	Male ICR mice	50, 100, or 200 mg kg^−1^ for 28 days	Aerobic endurance (running time to exhaustion)	Running time extended by 46%	By increasing Citrate synthase activity, the ability of muscles to oxidize fat is enhanced, thereby reducing the consumption of muscle glycogen	(118)
HP	*Hypericum perforatum* L.	Male ICR mice	2 mg kg^−1^ day^−1^ for 6 weeks	Aerobic endurance (swimming time to exhaustion)	Significant increase in the time to exhaustion during swimming	Reduces the accumulation of metabolites, enhances antioxidant capacity by regulating the AMPK/p53/Nrf2 signaling pathway, and inhibits the NF-κB signaling pathway to exert anti-inflammatory effects	(119)
SSO	*Hippophae rhamnoides* L.	Male ICR mice	0.85, 1.68, or 3.35 g kg^−1^ for 10 days	Aerobic endurance (swimming time to exhaustion)	Significant increase in the swimming time to exhaustion	Alleviates oxidative damage to the liver and the oxidative stress state of the central nervous system, improves the fatigue state of the central nervous system, inhibits inflammatory responses, and increases liver glycogen content	(120)
ECD	*Cistanche deserticola* Y. C. Ma	Male ICR mice	0.25, 0.50, or 1.00 g kg^−1^ day^−1^ for 3 weeks	Aerobic endurance (swimming time to exhaustion)	Significant increase in the time to exhaustion during swimming	Reduces the accumulation of metabolites, enhances oxygen supply capacity, and increases energy reserves	(121)

#### 3.3.4 Terpenes and lignins

Terpenes and lignin compounds are among the most widely distributed substances in nature, possessing various biological and pharmacological activities. Research indicates that terpene substances such as ginsenoside Rg1 and ursolic acid, as well as lignin compounds like eleutheroside, have positive effects solely on enhancing exercise endurance. The mechanisms of ginsenoside Rg1 can be summarized in two aspects. On the one hand, ginsenoside Rg1 promotes the phosphorylation of Akt in the skeletal muscle, activating the PI3K/Akt pathway, facilitating the translocation of Nrf2 to the nucleus, and inducing the expression of antioxidant enzyme genes (111). On the other hand, it can enhance endurance by regulating EGFR and influencing the metabolism of taurine and mannose-6-phosphate (112). At the molecular level, ginsenosides mediate these effects primarily by activating the AMPK/SIRT1/PGC1-α axis to promote mitochondrial biogenesis, engaging the PI3K/Akt/mTOR pathway to support muscle protein anabolism, and suppressing NF-κB-driven inflammatory signaling to limit exercise-induced muscle damage. Eleutheroside enhances endurance time by activating the adrenal system, increasing cAMP levels, promoting fat oxidation metabolism, and reducing reliance on glycogen (113). Ursolic acid can significantly prolong the fatigue swimming time under load in mice by activating the AMPK and PGC1-α pathways in the skeletal muscle (114).

#### 3.3.5 Other typical extracts

Among other typical extracts, the most classic functional factor that can simultaneously enhance physical strength and endurance is caffeine. Research indicates that caffeine supplementation can significantly enhance exercise speed during endurance activities, while also reducing feelings of stress and pain, thereby simultaneously improving physical endurance (115). Caffeine increases the activity of the Na^+^–K^+^ pump to exert effects peripherally, enhancing the excitation–contraction coupling necessary for muscle contraction, which contributes to improved physical performance. In addition, by competitively binding to adenosine receptors A1 and A2, caffeine inhibits the effects of adenosine, thereby reducing pain and exertion sensations during exercise and enhancing physical strength and endurance. Similar to the effects of caffeine, Capsaicin (CAP) also promotes mitochondrial biogenesis and ATP production by activating TRPV1 receptors (116).

In studies focused solely on enhancing endurance, compounds such as lycopene, docosanol, hypericum (HP), sea buckthorn seed oil (SSO), and phenylethanoid-rich extract (ECD) have been shown to significantly prolong the exhaustion swimming time of mice. Lycopene primarily promotes the transformation of skeletal muscle slow-twitch fibers through the AMPK pathway, enhancing the activity of the electron transport chain complexes and improving mitochondrial biogenesis and function (117). Docosanol increases the activity of citrate synthase, thereby enhancing the fat oxidation capacity of muscles and improving endurance (118). HP (119), SSO (120), and ECD (121) mainly enhance antioxidant capacity by regulating the AMPK/p53/Nrf2 signaling pathway. Furthermore, it has been found that β-carotene significantly increases maximum voluntary contraction, with mechanisms potentially involving the promotion of protein synthesis, improvement of energy metabolism, and reduction of oxidative damage (122). The studies of plant-derived functional factors regarding their effects and mechanisms for enhancing physical strength and endurance are summarized in Table 6.

## 4 Enhancement of physical strength and endurance: functional factors, action targets, and signaling pathways

The preceding sections have catalogued a wide range of nutritional bases and natural extracts that influence physical strength and endurance. To provide a coherent mechanistic framework, this section organizes the modes of action of these functional factors into distinct physiological categories.

### 4.1 Mechanisms for enhancing physical strength

#### 4.1.1 Increasing blood flow delivery efficiency

**Figure 1:**
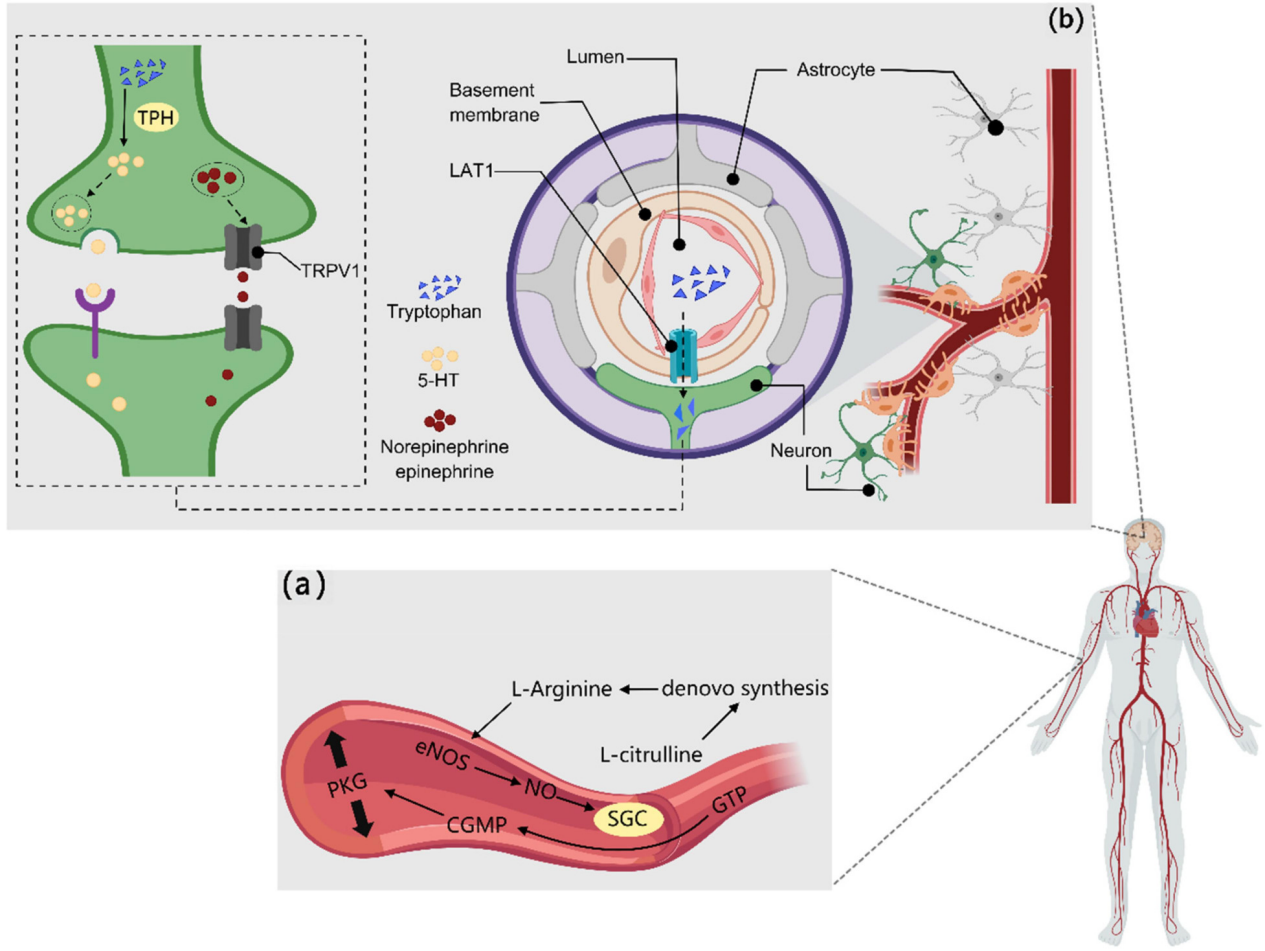
**(a)** Mechanism diagram of L-Arg and LC for vasodilation; **(b)** tryptophan transport mechanism. Tryptophan is transported from the blood across the blood–brain barrier to the brain, forming the pathway for 5-HT. In addition, there is a pathway for adrenaline to be transmitted through the TRPV1 receptor.

Increasing blood flow delivery efficiency is an important mechanism for enhancing physical strength, positively impacting the delivery of oxygen and nutrients, improving the clearance of metabolic waste, and enhancing muscle oxidative capacity. The increase in blood flow delivery efficiency is often accompanied by vasodilation (such as the effect of nitric oxide). As shown in Figure 1a, the soluble guanylyl cyclase (sGC)–cyclic guanosine monophosphate (cGMP) pathway is a signaling pathway centered around NO–sGC–cGMP, playing a crucial role in regulating vascular relaxation, neural signal transmission, metabolic balance, anti-inflammation, and cellular protection. Nitric oxide (NO) activates the sGC–cGMP signaling pathway, reducing intracellular calcium ion concentration in smooth muscle cells, leading to relaxation of the vascular smooth muscle and vasodilation, thereby increasing blood flow (128). NO is catalyzed by nitric oxide synthase (NOS), including endothelial NOS (eNOS) and neuronal NOS (nNOSμ). Among these, nNOSμ is mainly found in the skeletal muscle and neural tissue, responsible for regulating local blood flow, while eNOS is primarily located in vascular endothelial cells, mainly responsible for regulating systemic blood flow (129). For example, it was found that L-Arg is converted into NO and LC under the action of NOS. LC can be converted back to L-Arg through *de novo* synthesis in the kidneys. Since LC is not easily broken down by arginase, it can provide more substrate for NOS, thereby increasing NO production (130). Blood flow delivery efficiency may also be related to the loading capacity of blood, such as the oxygen-carrying capacity of red blood cells. Research has shown that iron supplementation may improve the iron content within red blood cells, enhancing their ability to carry oxygen (54). Increasing blood flow delivery efficiency can ensure that more oxygen reaches active skeletal muscles, thereby supporting mitochondrial oxidative metabolism and enhancing ATP production.

#### 4.1.2 Reducing fatigue in the central nervous system

Reducing fatigue in the central nervous system is an important mechanism for enhancing physical performance, with its core focus on regulating the levels of neurotransmitters (such as 5-HT, norepinephrine, and dopamine) and neuromodulators (such as adenosine) to maintain arousal, attention, and motivation during exercise (131). Neurotransmitters can exert either inhibitory or facilitatory effects on physical performance. For instance, 5-HT is a neurotransmitter associated with mood and behavioral inhibition, and elevated levels can lead to increased feelings of fatigue, a phenomenon known as the “central fatigue hypothesis.” As shown in Figure 1b, 5-HT in the brain is metabolized from tryptophan through tryptophanase (TYH). During exercise, the breakdown of fat releases the tryptophan carrier (albumin), leading to an increase in free tryptophan levels in blood. This elevated level crosses the blood–brain barrier via the LAT1 transporter, resulting in an increase in 5-HT levels in the brain (132). Therefore, reducing the production of 5-HT in the brain is an important method to alleviate central nervous system fatigue. For example, BCAAs reduce the entry of tryptophan by competing with it for LAT1 in the blood–brain barrier, thereby inhibiting the excessive production of 5-HT (133). In addition, royal jelly may further reduce the production of 5-HT by regulating the activity of TYH (67). Apart from lowering 5-HT levels, promoting the secretion of excitatory neurotransmitters (such as norepinephrine and dopamine) is also vital for reducing central nervous system fatigue. For instance, capsaicin activates TRPV1 receptors, increasing the secretion of norepinephrine and epinephrine, which not only enhances mental concentration but also helps maintain thermal balance during exercise by regulating body temperature (116). Although the targets and mechanisms of these processes differ, their core objective is consistent: to effectively enhance physical performance and delay the onset of fatigue by reducing inhibitory signals in the central nervous system, enhancing excitatory neural signals, and improving metabolic regulation.

#### 4.1.3 Maintenance of calcium homeostasis between mitochondria and endoplasmic reticulum

**Figure 2:**
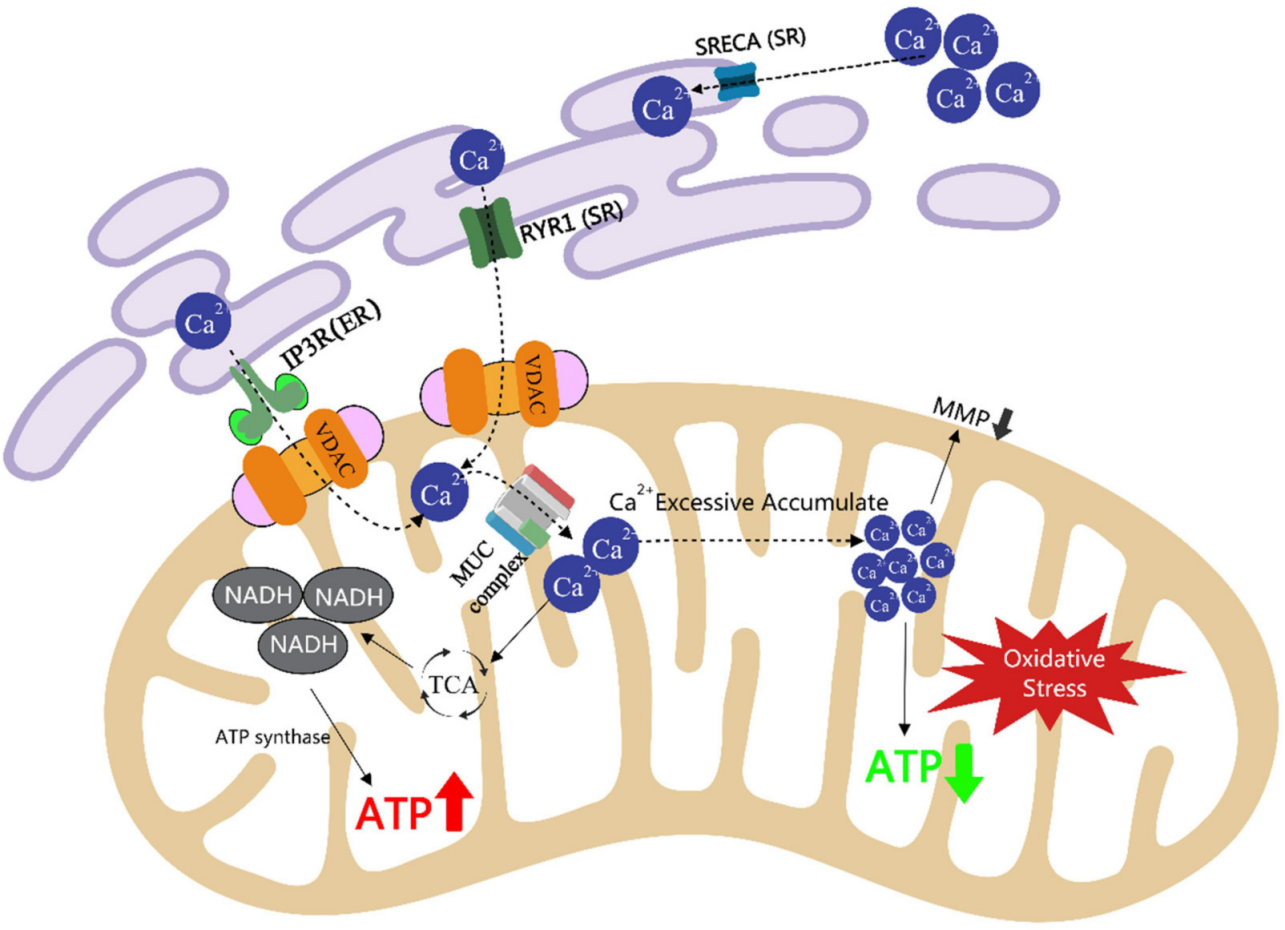
Calcium ion regulation mechanism. The endoplasmic reticulum or sarcoplasmic reticulum is involved in the pathways of calcium ion release or uptake, as well as the positive and negative roles of calcium ions in mitochondria.

Maintaining endoplasmic reticulum (ER) calcium homeostasis plays a crucial role in enhancing physical performance, primarily by regulating calcium ion transfer and dynamic equilibrium to optimize energy metabolism, sustain mitochondrial function, and alleviate oxidative stress. The ER and mitochondria form physical contacts through MAMs, which play a central role in calcium ion transport. Maintaining calcium homeostasis is essential for mitochondrial energy metabolism and antioxidant function. As shown in Figure 2, muscle cells maintain calcium homeostasis by enhancing sarcoplasmic reticulum (SR) SERCA activity to accelerate calcium reuptake. Calcium release in these cells depends on the RYR1 channel, after which calcium ions enter the mitochondria through the VDAC1–MCU pathway. In contrast, non-muscle cells mediate calcium release through the IP3R in the endoplasmic reticulum, sharing the same mitochondrial calcium transport mechanism. Appropriate levels of calcium entering the mitochondria stimulate the TCA cycle and NADH formation, thereby stimulating respiratory chain activity and increasing ATP production, ultimately enhancing energy output (134). However, an imbalance in calcium homeostasis can lead to mitochondrial calcium overload, triggering the accumulation of ROS and a decrease in MMP, ultimately impairing muscle function (135). By examining the mechanisms of action of *Polygonatum sibiricum* Redouté polysaccharide, VD, and selenium, it can be observed that they regulate calcium homeostasis through different pathways, ultimately enhancing physical strength. *Polygonatum sibiricum* Redouté polysaccharide reduces the excessive expression of MAM-related proteins induced by D-gal, decreases the excessive transport of calcium ions from the endoplasmic reticulum to the mitochondria, restores calcium homeostasis, and alleviates oxidative stress (99). VD enhances the sensitivity of the sarcoplasmic reticulum to calcium, promoting calcium release and reuptake, thereby improving the efficiency of calcium signaling and enhancing muscle contraction capability (63). Selenium protects the function of the RyR1 calcium channel by upregulating the expression of the antioxidant protein Selenoprotein N, while enhancing the activity of SERCA, thereby maintaining calcium homeostasis and improving muscle function (52). There are various ways to maintain calcium homeostasis, including regulating the expression of calcium channel proteins, optimizing calcium signaling, and alleviating oxidative stress.

### 4.2 Mechanism for enhancing endurance

The three mechanisms described in Section 4.1 all contribute primarily to acute force production and physical strength. Improving endurance requires a different set of adaptations centered on oxidative metabolic capacity and fatigue resistance over prolonged effort.

**Figure 3:**
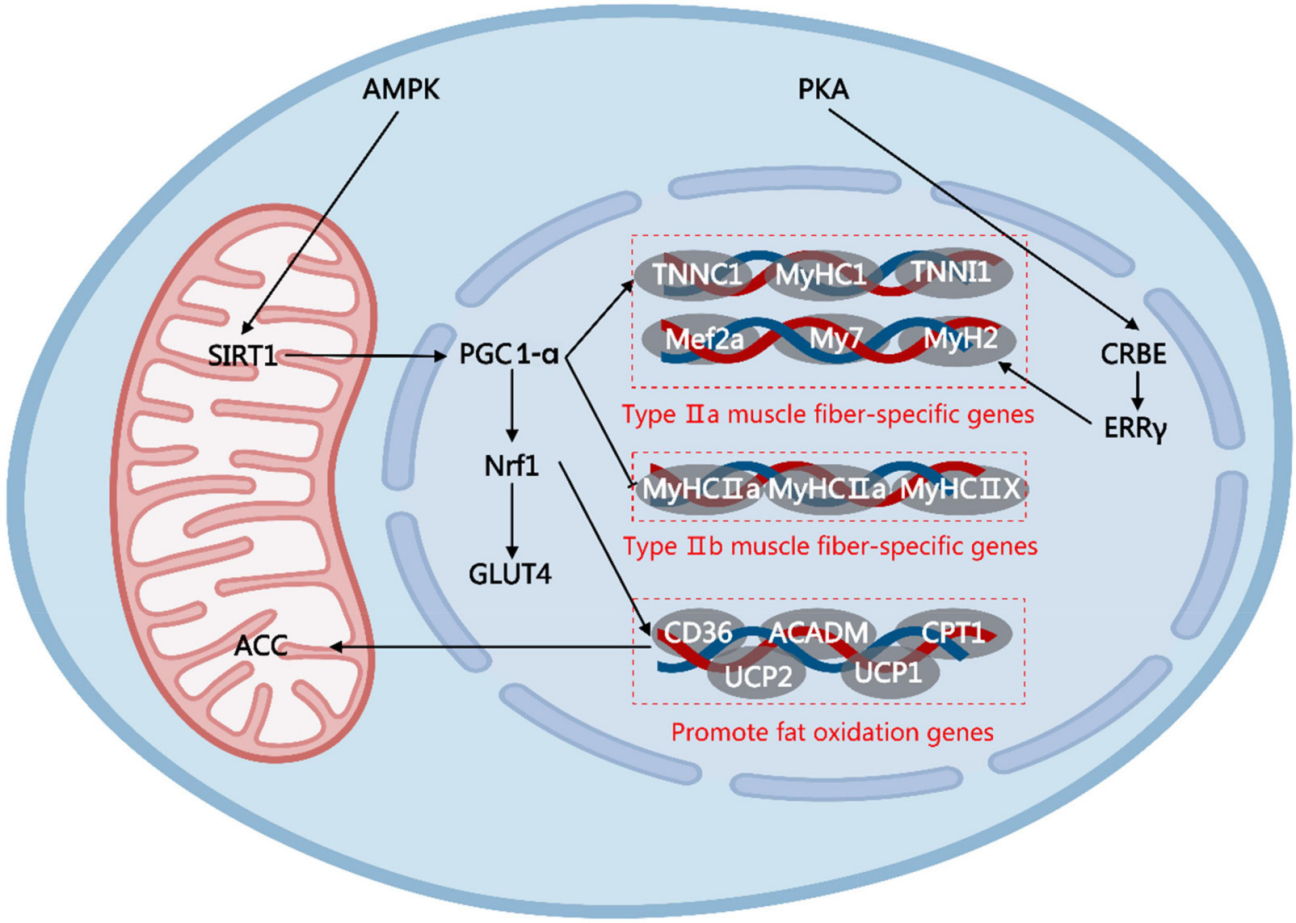
Pathways and related genes for increasing the proportion of type IIa muscle fibers.

Increasing the proportion of type IIa muscle fibers is a key mechanism for improving endurance, as these fibers possess a robust oxidative metabolic capacity and high fatigue resistance, which can significantly enhance the ability and fatigue resistance during prolonged low-intensity exercise. Type IIa muscle fibers require a sustained and stable energy supply to perform prolonged work, and reliance solely on the anaerobic glycolysis of type IIb muscle fibers cannot meet this demand. Therefore, type IIa fibers are highly dependent on the mitochondrial oxidative metabolic system (136). As shown in Figure 3, the regulation of the transition from fast-twitch muscle fibers to slow-twitch muscle fibers or the increase in the proportion of slow-twitch muscle fibers mainly emanates from two signaling pathways, one of which is the AMPK/SIRT1/PGC1-α pathway. For example, lycopene promotes mitochondrial biogenesis and fatty acid oxidation by activating PGC1-α, thereby indirectly increasing the proportion of type IIa muscle fibers. It also enhances the expression of type IIa muscle fiber–specific genes, including MyHC I, Troponin I1 (TNNI1), and Troponin C1 (TNNC1), and inhibits the expression of type IIb muscle fiber–specific genes (*MyHC IIa*, *MyHC IIx*, *MyHC IIb*) by binding to various transcription factors such as NRF1 and MEF2 (117). The second is the PKA–CREB–ERRγ pathway. For example, the extract of *L. barbarum* directly promotes the conversion of muscle fiber types by activating CREB and upregulating the expression of ERRγ, which facilitates the transcription of its downstream genes (*MEF2*, *Myh IIx*) to form type IIa muscle fibers (84). The roles of the two pathways differ slightly: PGC1-α is more prominent in inhibiting type IIb muscle fiber–specific gene expression and enhancing type IIa muscle fiber–specific gene expression, while ERRγ primarily promotes muscle gene expression, with a relatively weak inhibition of type IIb muscle fiber–specific genes. In addition, several type IIa muscle fiber–specific genes (such as *MyHC I* and *MyHC IIa*) may be coregulated by both signaling pathways, but the AMPK/SIRT1/PGC1-α pathway focuses more on indirectly supporting the formation of type IIa muscle fibers through mitochondrial synthesis and metabolic regulation, whereas the PKA-CREB pathway mainly regulates the expression of type IIa muscle fiber–specific genes directly through ERRγ.

### 4.3 Mechanism for enhancing physical strength and endurance

In addition to the mechanisms that selectively support either strength or endurance, a number of functional factors act through pathways that simultaneously benefit both outcomes. These shared mechanisms reflect the interconnected nature of muscular physiology, in which oxidative capacity, metabolic efficiency, inflammatory control, and gut health all contribute to overall physical performance.

#### 4.3.1 Reducing oxidative stress and decreasing metabolite accumulation

Reducing the accumulation of metabolites and decreasing oxidative stress is one of the mechanisms of most functional factors. After exercise, the body produces many metabolic by-products, and the elevated levels of these metabolites not only reflect the extent of muscle damage and fatigue but may also have adverse effects on the body (137). For example, muscle injury is primarily assessed through the blood levels of CK, ALT, and AST, while fatigue indicators include the levels of BLA, BUN, and LDH, among others. The excessive accumulation of metabolic by-products may lead to the production of certain inflammatory responses; for instance, BLA can cause acidosis, interfering with ATP production and muscle function (138). The production of oxidative stress product MDA will also exacerbate muscle damage (139). Most functional factors that enhance physical endurance do so indirectly by positively regulating the levels of metabolic products.

The reduction of metabolite accumulation is closely related to the decrease of oxidative stress. Oxidative stress refers to ROS produced after exercise, such as hydroxyl radicals and superoxide anions, which cause lipid peroxidation damage to the skeletal muscle and liver mitochondria (140). The body has an endogenous antioxidant enzyme system, including SOD, GSH-Px, and CAT, which is used to eliminate ROS (141). When ROS exceed the clearance capacity of the antioxidant system, oxidative stress occurs, leading to the damage of macromolecules and organelles. A typical example is the impairment of the sodium–potassium pump in cells. For instance, anthocyanins and NAC maintain the activity of the sodium–potassium pump through their own antioxidant capabilities, demonstrating the effects of enhanced physical strength and endurance, respectively (25, 105). To reduce the accumulation of metabolites and decrease oxidative stress, one can enhance antioxidant capacity by increasing the levels of SOD, GSH-Px, and CAT. In addition, the regulation of the key antioxidant factor Nrf2 can be employed. During oxidative stress, by controlling the separation of Nrf2 from Keap1, Nrf2 can enter the cell nucleus and bind to the antioxidant response element (ARE), thereby regulating the expression of various antioxidant enzyme genes (142).

#### 4.3.2 Regulatory capacity of metabolism

**Figure 4:**
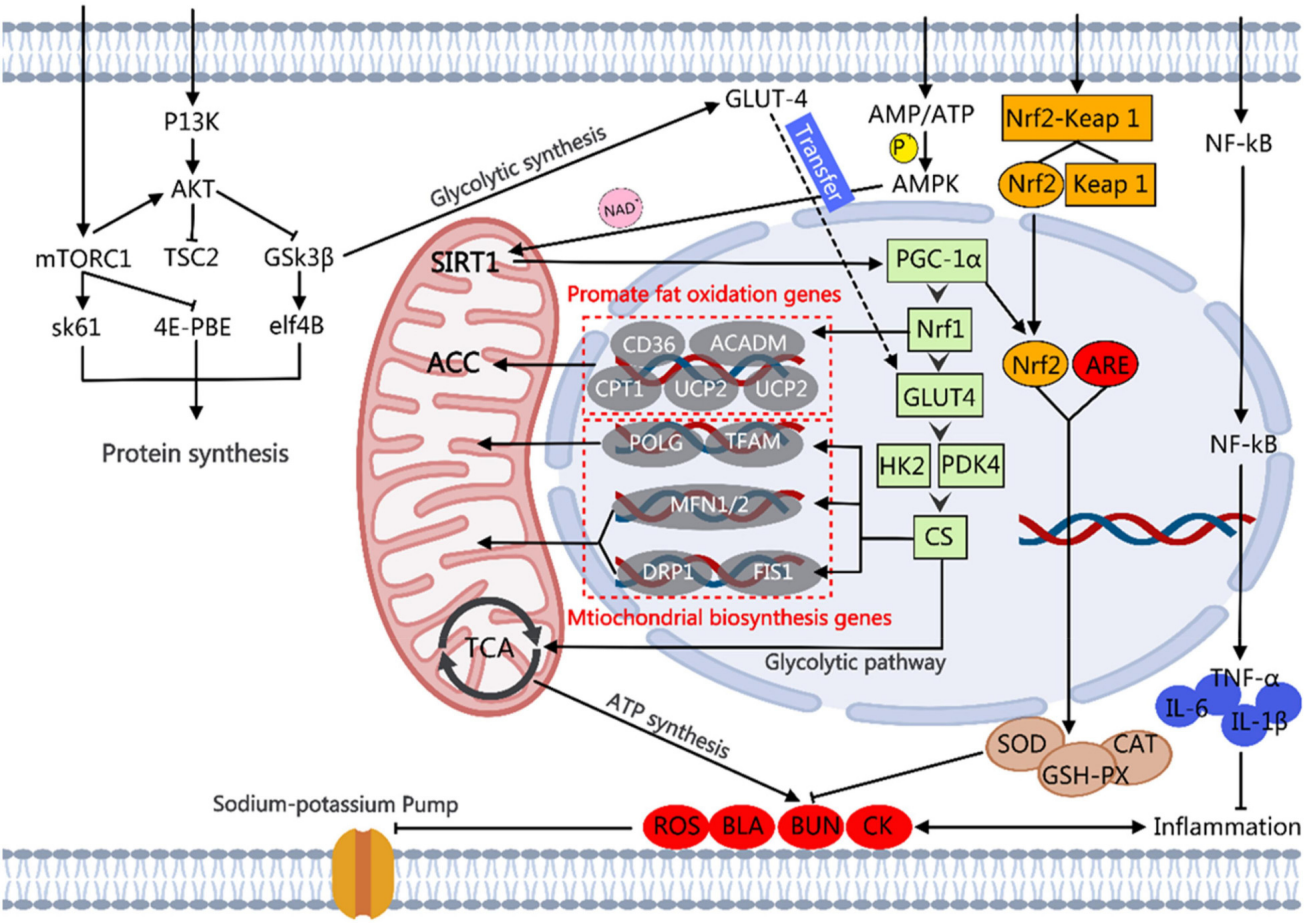
Potential mechanisms for enhancing physical strength and endurance, including protein synthesis pathways, glycogen storage and glycolysis pathways, lipolysis pathways, metabolite production pathways, as well as mechanisms for enhancing antioxidant capacity and immunity.

Regulating energy metabolism is the primary mechanism for increasing physical endurance, which mainly includes enhancing the storage of glycogen or protein, promoting lipid or glycogen metabolism, enhancing mitochondrial biogenesis, and repairing mitochondrial dysfunction. At a macro level, the improvement of endurance and physical strength relies on the synergistic action of multiple energy systems. The enhancement of endurance primarily depends on glycogen or protein stored in the liver and muscles as important energy sources. When blood sugar levels drop, liver glycogen can quickly convert to glucose and be released into the bloodstream. The enhancement of physical strength, on the other hand, provides additional energy through fat oxidation while delaying glycogen depletion. Furthermore, the quantity and function of mitochondria can directly determine the oxidative phosphorylation efficiency of the skeletal muscle (143). At the molecular level, these energy metabolism processes are closely related to the key regulatory factors mTOR and AMPK. As shown in Figure 4, mTOR is a critical factor in protein and glycogen synthesis, and it can be activated directly or through the activation of mTOR by PI3K and Akt (or by inhibiting TSC2, which positively regulates mTORC1 activity). This activation subsequently enhances translation efficiency and promotes protein synthesis by phosphorylating S6K and activating the translation factor eIF4B (144). The PI3K/Akt pathway can accelerate the entry of glucose from the bloodstream into muscle and fat cells by promoting the membrane translocation of GLUT4. It restores the activity of glycogen synthase by inhibiting the activity of GSK-3β, thereby promoting glycogen storage while simultaneously relieving the inhibitory effect on eIF2B, which facilitates the entire protein synthesis process (145). Through this mechanism, for example, chicken essence enhances physical strength (69), but the aqueous extract (85) and *A. bidentata* polysaccharides (94) enhance endurance (69).

As shown in Figure 4, AMPK can promote fatty acid oxidation and glycolysis, while SIRT1 can directly regulate the activity of PGC1-α through phosphorylation and deacetylation and indirectly regulate the expression or activity of NRF1. This pathway also constitutes a key regulatory axis for mitochondrial biogenesis. Specifically, the promotion of fatty acid oxidation mainly occurs through the upregulation of target genes such as uncoupling protein 1 (*UCP1*), which increases the rate of fatty acid utilization. Uncoupling protein 2 (*UCP2*) optimizes mitochondrial function. Carnitine palmitoyltransferase 1 (CPT1) facilitates the transport of fatty acids into the mitochondria and acyl-CoA dehydrogenase, C-4 to C-12 straight chain (ACADM), which accelerates the rate of fatty acid oxidation, and into cluster of differentiation 36 (CD36), which enhances fatty acid uptake, thereby promoting the efficient oxidation of fatty acids (146). At the same time, it indirectly regulates ACC activity, achieving a high degree of coordination between fatty acid oxidation and mitochondrial energy metabolism. Through this mechanism, for example, L-carnitine enhances physical strength, and *D. officinale* polysaccharides enhance endurance (19). To promote glycolysis, it primarily enhances the transport of GLUT4 to the cell membrane, thereby increasing glucose uptake. Glucose is metabolized to pyruvate via hexokinase 2, and the regulation of pyruvate dehydrogenase kinase 4 (PDK4) maintains the conversion of pyruvate to acetyl-CoA, facilitating its entry into the TCA cycle. Citrate synthase (CS) catalyzes the reaction between acetyl-CoA and oxaloacetate to produce citrate, officially initiating the TCA cycle. The acetyl-CoA that enters the TCA cycle is efficiently utilized, and the products of glycolysis are completely oxidized, releasing a significant amount of energy (147).

For mitochondrial metabolism and mitochondrial biogenesis, as shown in Figure 4, there are two mechanisms originating from the AMPK/SIRT1/PGC1-α signaling pathway. First, it regulates mitochondrial generation through molecular signal transduction: CS serves as an important bridge by activating the expression of the DNA polymerase gamma (*POLG*) gene, increasing the copy number of mtDNA, and supporting mitochondrial protein synthesis. By promoting the expression and activity of TFAM, it enhances the transcription efficiency and stability of mitochondrial DNA (148). Second, it promotes the structural remodeling of mitochondria by regulating the dynamic balance of mitochondria: by facilitating fusion (upregulating *MFN1/2* genes), it maintains a healthy mitochondrial network, providing a stable template and requirements for mitochondrial biogenesis. By moderately inhibiting fission (downregulating *DRP1/FIS1* genes), it avoids the excessive production of harmful fragmented mitochondria due to fission, thereby optimizing the mitochondrial pool (149). Some functional factors that enhance physical strength can also increase the synthesis of phosphocreatine in the mitochondria, with creatine being the most typical example. Research has shown that creatine is primarily stored in the mitochondria in the form of phosphocreatine. During high-intensity exercise, compared with the ATP produced through oxidative phosphorylation and anaerobic glycolysis, the supplementation of phosphocreatine allows ADP to be converted into ATP more quickly and effectively, providing ample energy for muscle and protein synthesis (13).

#### 4.3.3 Enhance immunity and anti-inflammation

Acute inflammation and chronic inflammation are subsequent symptoms induced by oxidative stress during excessive muscle exercise, manifested by an increase in proinflammatory cytokines in the skeletal muscle and plasma. Enhancing immune and anti-inflammatory responses is one of the mechanisms for improving physical endurance function factors (150). During exercise, the excessive release of proinflammatory cytokines such as TNF-α, IL-1β, and IL-6 can impair bodily functions and induce fatigue. The body can alleviate the inflammatory response by negatively regulating the activity of these proinflammatory factors through the anti-inflammatory cytokine IL-10 (151). NF-κB, as a key factor in the release of proinflammatory cytokines, experiences increased phosphorylation after intense exercise, leading to a vicious cycle of inflammatory response and mitochondrial dysfunction (152). Some functional factors achieve anti-inflammatory and immune effects by inhibiting NF-κB. Through this mechanism, for example, curcumin can enhance physical strength (110), but CL (77) extract and polysaccharide from *P. heterophylla* (Miq.) Pax ex Pax et Hoffm (95) can enhance endurance.

#### 4.3.4 Enhance intestinal health

There is a high correlation between changes in the composition of the body and gastrointestinal microbiota during exercise, and the gut microbiota can regulate metabolic homeostasis through its coding of metabolic enzymes (153). The research identified three main mechanisms: first, improving the ratio of gut microbiota. For example, lactic acid bacteria can reduce the *Firmicutes*/*Bacteroidetes* ratio in the gut, which is associated with obesity and metabolic disorders (68). By increasing this ratio, physical strength is enhanced; second, increasing the proportion of probiotics and reducing the proportion of harmful bacteria. Typical probiotics such as *Lactobacillus* and *Bifidobacterium* can improve metabolic efficiency and reduce oxidative stress and inflammation (154), and the proliferation of harmful bacteria (such as *Enterococcus* and *Streptococcus*) can produce toxins (such as hemolysins and cytotoxins) or stimulate the intestinal immune system, leading to local or systemic inflammatory responses (155). This regulatory effect is usually accompanied by an increase in the production of short-chain fatty acids (such as acetate, propionate, and butyrate), further optimizing the intestinal environment. Short-chain fatty acids are products of the fermentation of dietary fibers and other substrates by gut microbiota, participating in energy metabolism, reducing inflammation, and enhancing intestinal barrier function, thereby indirectly improving exercise endurance (156). Konjac polysaccharide (96) and hawthorn berry polyphenols (104) enhance endurance through this mechanism. Thirdly, gut microbiota regulation exerts its biological effects by modulating the gut–liver–muscle axis. This mechanism increases the production of short-chain fatty acids and reduces the generation of trimethylamine (TMA), which is produced by gut bacteria through the metabolism of nitrogen-containing compounds (such as choline and carnitine). TMA then enters the liver and is oxidized to trimethylamine N-oxide (TMAO) (157). In the intestines, TMAO disrupts the intestinal mucosal barrier, allowing toxins to enter the bloodstream. In the liver, TMAO can increase oxidative stress levels, exacerbating liver inflammation and metabolic burden. TMAO may decrease the efficiency of muscle cells in utilizing nutrients, affecting muscle metabolic adaptability and exercise capacity (158). A study found that the extract of astragali radix extends endurance by reducing the production of trimethylamine (95).

## 5 Limitations and future work

This review provides a comprehensive overview of the effects and mechanisms of various functional factors on enhancing physical endurance and several directions worthy of further exploration.

When studying the impact of functional factors on athletic performance, the differences in physiological and biochemical responses among individuals are an important factor that cannot be overlooked. Although taking caffeine for enhancing endurance performance is widely recognized, there is also a substantial body of research indicating that caffeine does not consistently and positively affect the athletic performance of everyone. This variability in individual responses may stem from multiple factors, including genetics, metabolism, and physical constitution, highlighting the necessity for further in-depth research (159). Therefore, future studies need to be more systematic in exploring the mechanisms of action, response intensity, and potential effects of different functional factors in various individuals, in order to establish more precise personalized sports nutrition and supplementation strategies.

In the study of exploring the mechanisms by which specific substances affect physical strength and endurance, it was found that some research has limitations in terms of conceptual definitions and experimental design. Although the activation of mitochondrial biogenesis was observed through the AMPK–PGC1-α–NRF1–TFAM signaling pathway, which resulted in extending running time and improved hanging performance in mice, there was no direct assessment of the specific enhancement of physical strength. This primarily stems from the failure of research to clearly distinguish between the concepts of “physical strength” and “endurance,” as well as the lack of targeted testing indicators and systematic experimental design. Consequently, this has led to an inability to comprehensively evaluate the actual impact of mitochondrial biogenesis on physical strength. Therefore, subsequent research needs to adopt more refined and comprehensive experimental protocols (101). In addition, there are many potential functional factors that have not been included in this study. For example, Schisandrin B (160) and Herba Cistanches extract (161) have been shown to protect the brains, hearts, livers, and skin tissues of rodents from oxidative stress by enhancing mitochondrial antioxidant status. However, there is no research indicating their specific effects on enhancing physical endurance. It was found that blood flow increased after supplementation; however, it was observed that plasma NO*_x_* levels did not increase. This is at variance with the common belief that consuming nitrate-rich foods would enhance the body's production of NO and elevate NO*_x_* levels (90). Because of the transient and short-lived nature of NO production and its effects, the timing of measuring plasma NO*_x_* levels in studies may fail to capture the brief increase in NO levels following supplementation. Furthermore, its vasodilatory effect may not solely depend on the increase in NO but could also involve other vasoactive substances, such as the reduction of angiotensin II or endothelin-1 activity, which promote vasodilation (162).

Utilizing omics technologies and systems biology approaches may help reveal deeper mechanisms of action. For instance, proteomics techniques can be employed to comprehensively analyze changes in protein phosphorylation levels within the AMPK and PGC1-α signaling pathways, among others. For complex functional factors such as plant extracts and multiomics (genomics, transcriptomics, proteomics, metabolomics), an integrative analysis can be conducted to construct molecular networks of their actions and identify key regulatory nodes.

Different functional factors exhibit synergistic effects. Existing evidence indicates that simultaneous supplementation with royal jelly and coenzyme Q10 can enhance physical endurance (163). Future research can focus on exploring the optimal ratios and combination strategies of different functional factors. For instance, substances that regulate energy metabolism, such as BCAAs and L-carnitine, may have a synergistic effect when used together in promoting fat oxidation and delaying glycogen depletion. Similarly, combinations of central nervous system regulators such as caffeine and rhodiola rosea glycosides are expected to achieve more sustained antifatigue effects and may reduce the potential side effects associated with high doses of individual substances. Such studies not only require a systematic examination of dose–response relationships and time dynamics but must also fully consider individual differences, long-term safety, and the specificity of different types of exercise. Through multidimensional and precise research methods, scientific evidence can be provided for personalized sports nutrition supplementation.

## 6 Conclusion

The evidence analyzed in this scoping review clearly supports the positive effects of functional factors on physical strength and endurance, although the quality of evidence varies significantly across different categories. For functional factors supported by sufficient human evidence, stratified application can be made according to their primary effects. Those applicable to strength-type sports include creatine, HMB, VD, L-arginine, LC, L-carnitine, curcumin, epicatechin, and golden root extract, with mechanisms encompassing phosphocreatine resynthesis, mTOR pathway activation, calcium homeostasis regulation, and nitric oxide–mediated vasodilation. Those applicable to endurance-type sports include β-alanine, taurine, iron, caffeine, eleutheroside, anthocyanins, powdered Montmorency tart cherry, and beetroot concentrate, with mechanisms encompassing the buffering of exercise-induced acidosis, enhancement of oxygen-carrying capacity, and antagonism of adenosine receptors. Those applicable to mixed-discipline sports include branched-chain amino acids, glutamine, whey protein and other plant-based proteins, coenzyme Q10, and magnesium, all of which share the multipathway characteristic of promoting muscle protein synthesis while improving oxidative metabolism. The above functional factors all possess well-defined mechanisms of action, clearly established effective dose ranges, and documented safety profiles and can therefore be directly applied in evidence-based sports nutrition practice. For the broader category of natural extracts, including plant-derived polysaccharides, polyphenols and flavonoids, terpenes and lignins, and complex extracts, as well as microbial-derived fungal polysaccharides and mycelium-derived bioactive components, and animal-derived bioactive components such as royal jelly, *C. elaphus* L. extract, and chicken essence, stable and reliable mechanisms for enhancing physical strength and endurance have been demonstrated in preclinical research models, yet sufficient human clinical trial validation has not been obtained. Pending the accumulation of adequate clinical evidence, these extracts should not be recommended as equivalent to verified functional factors.

In summary, the evidence integrated in this scoping review indicates that functional factors can improve physical strength and endurance through eight core physiological pathways via diverse regulatory mechanisms, encompassing central nervous system regulation, improvement of blood circulation, maintenance of calcium homeostasis, increase in the proportion of type IIa muscle fibers, reduction of oxidative stress and metabolite accumulation, optimization of energy metabolism, enhancement of immunity and anti-inflammation, and improvement of intestinal health. Functional factors with a nutritional basis operate predominantly through energy metabolism optimization, calcium homeostasis maintenance, and regulation of protein synthesis, whereas functional factors derived from natural extracts exert their effects more through multitarget synergistic mechanisms, including antioxidant and anti-inflammatory action, immune enhancement, gut microbiota regulation, and muscle fiber type transformation. By integrating pharmacological theory with modern signaling pathway analysis within a unified functional classification system, this review provides a scientific foundation for personalized, targeted, and evidence-graded sports nutrition strategies. For well-characterized functional factors with clear mechanisms and sufficient evidence, the corresponding nutrition strategies are already ready for direct practical application, and as clinical research data on natural extracts continue to accumulate in the future, their scope of application will be further expanded.
